# Supplementary material for: China’s electric vehicle and climate ambitions jeopardized by surging critical material prices
Source: Nat Commun. 2023 Mar 4;14:1246. doi: 10.1038/s41467-023-36957-4 (PMC9985616; doi:10.1038/s41467-023-36957-4)
Supplement: Supplementary file 1 — Suplementary information [file 41467_2023_36957_MOESM1_ESM.pdf]

Supplementary Information *for*

China's Electric Vehicle and Climate Ambitions Jeopardized by Surging Critical Material Prices

Hetong Wang,[1,10] Kuishuang Feng,[2,10] Peng Wang,[3,7,8,*] Yuyao Yang,[4] Laixiang Sun,[2,9,*] Fan Yang,[5] Wei-Qiang Chen,[3,8] Yiyi Zhang,[6] Jiashuo Li[1,*]

[1] Institute of Blue and Green Development, Shandong University, Weihai 264209, China

[2] Department of Geographical Sciences, University of Maryland, College Park, MD 20742, USA

[3] Key Lab of Urban Environment and Health, Institute of Urban Environment, Chinese Academy of Sciences, Xiamen 361021, China

[4] Guanghua School of Management, Peking University, Beijing 100871, China

[5] Department of Planning, Aalborg University, 9000 Aalborg, Denmark

[6] Guangxi Key Laboratory of Intelligent Control and Maintenance of Power Equipment, Guangxi University, Nanning 530004, China

[7] Ganjiang Innovation Academy, Chinese Academy of Sciences, Ganzhou 341000, China;

[8] University of Chinese Academy of Sciences, Beijing 100864, China

[9] School of Finance & Management, SOAS University of London, London WC1H 0XG, UK

[10] These authors contributed equally

*Correspondence: pwang@iue.ac.cn (P.W.), LSun123@umd.edu (L.S.), lijiashuo@sdu.edu.cn (J.L.)

Supplementary Notes S1-S6
Supplementary Tables S1-S8
Supplementary Figures S1-S45

Supplementary Notes

Note S1. Calculation principles in GCAM

The detailed description of Global Change Assessment Model (GCAM) approaches can be found in the website of Joint Global Change Research Institute (JGCRI, https://gcims.pnnl.gov/modeling/gcam-global-change-analysis-model). For a clear information, we have illustrated the transportation module in Fig. S2. The core functions for our quantification of transportation service are given as follows:

1.1 Demand for transportation service

Demand for passenger transportation (D_P, in passenger-km) in region r and future time period t is estimated with the following equation:

$$D_{P}^{r,t} = \delta^{r}(Y_{I}^{r,t})^{\alpha}(P_{I}^{r,t})^{\beta}(N_{I}^{r,t}) \tag{1}$$

Where δ is a base year calibration parameter; Y_I is the index for income in the form of per-capita GDP at time t divided by the per-capita GDP in the base year; P_I is the index of the price of transportation aggregated across all modes, size classes, and technologies and calculated as the ratio of price in time t to the price in the base year; N_I is the population in region r, in time t. Finally, α and β are income and price elasticities, respectively, with respect to per capita passenger demand, and are estimated by regressions based on historical demand data.

Demand for freight transportation (D_F, in tonne-km) in region r and future time period t is estimated with a very similar equation:

$$D_{F}^{r,t} = \delta^{r}(GDP_{I}^{r,t})^{\alpha'}(P_{I}^{r,t})^{\beta'} \tag{2}$$

Different from the passenger transportation sector, the demand of the freight transportation sector is estimated on a per capita basis and then aggregated across the entire population. Therefore, income is expressed by the index of total GDP. The income and price elasticities of freight demand are designated by α' and β' respectively.

1.2 Generalized cost of transportation service

The total generalized cost of transportation services (P, in \$/PKT or \$/Tonne-KM) is derived as the weighted average cost of each available mode:

$$P^{r,t} = \sum_{i}(S^{i})(P^{i,r,t}) \tag{3}$$

where S^i is the share of mode i in terms of passenger-KM or Tonne-KM.

The costs by mode are calculated as the weighted average costs of all constituent size classes plus the time value costs (value of travel time) associated with the mode, which is presented as follows:

$$P^{i,r,t} = P^{i,r,t}_{time} + \sum_i (S^s)(P^{s,i,r,t}) \tag{4}$$

where S^s is the share of size class (*s*) under mode (*i*) in terms of passenger-KM or Tonne-KM.

Time value costs are indicated as follows:

$$P^{i,r,t}_{time} = \delta^i + \frac{W^{r,t}}{Sp^{i,r,t}} \tag{5}$$

Where *W* is the wage rate ($/hour) calculated from the per capita GDP; S_p is the average door-to-door speed of mode *i* (KM/hour), which varies by mode, region and time; and δ is a unitless parameter representing the cost associated with travel expressed as a multiplier of the wage rate (value of time, or VOT).

The costs for each size class (*s*), in turn, are calculated as the weighted average costs of all constituent technologies (*j*):

$$P^{s,i,r,t} = \sum_i (S^j)(P^{j,s,i,r,t}) \tag{6}$$

Finally, technology costs may be broken down into fuel costs and non-fuel costs:

$$P^{j,s,i,r,t} = \frac{(P^{r,t}_{fuel})(EI^{j,s,i,r,t}) + P^{j,s,i,r,t}_{NF}}{LF^{i,r,t}} \tag{7}$$

Where, P_{fuel} is the fuel price ($/MJ), which is endogenous; *EI* is the vehicle energy or fuel intensity (MJ/VKT); P_{NF} is the non-fuel price of transportation for the given mode; and *LF* is the load factor defined either as passengers per vehicle or tonnes per vehicle.

The non-fuel costs are estimated for light-duty vehicles based on exogenous assumptions about vehicle capital costs (purchase cost, infrastructure cost, and others), non-fuel operating costs (maintenance cost, registration and insurance cost, and tolls), financing, and annual vehicle utilization (vehicle-km per year). For freight technologies and passenger bus, the non-fuel cost is estimated by deducting estimated fuel costs from total service costs (Capital Expenditure-CAPEX and non-fuel Operational Expenditure-OPEX). In either case, the non-fuel cost is converted to dollars per vehicle-km for the equation above. The model then computes market shares of the different technologies as described in the logit choice below.

1.3 Market shares of transportation types

The market share of each transportation mode is determined by a calibrated logit formulation, which is given by the following equation[1,2]:

$$S^{i,r,t} = \frac{(SW^{i,r})(P^{i,r,t})^{\lambda_i}}{\sum_i (SW^{i,r})(P^{i,r,t})^{\lambda_i}} \quad (8)$$

where S is the market share; SW is the share weight; P^i is the cost of transport service for a mode I; and λ is the logit exponent. The share weight is a calibration parameter, and the logit exponent regulates the degree to which future price changes will be reflected in model shifts.

Note S2. Critical material price forecast

There is great uncertainty in the long-term prediction of material prices. Therefore, we use three methods to do the forecasts. The historical price dynamics of these critical materials, as the foundation for the price forecasting, are shown in Fig. S3.

In the High scenarios, we postulate that the initial surge of demand for EV will accelerate the rising of the prices of critical materials, while the acceleration will dampen in the medium-run and the price level will become flatten in the long-run, due to the increased recycling and the increased use of substitutes. Thus, we apply a logistic function to predict the prices of critical materials, in which the relationship between price and demand quantity is an S-shaped curve lying between the lower and upper limit of the price (Eq. 9)[3]:

$$\frac{P-P_L}{P_U-P_L} = \frac{1}{1+exp(-c_1-c_2D)} \quad (9)$$

where P_L is the lower limit of material price, which is derived from historical data; P_U is the upper limit of material price, which we estimate to be multiple of the highest price observed in the history; D is the quantity of annual material demand; and c_1 and c_2 are coefficients, which are estimated by regressions based on historical demand data.

In the Medium scenarios, we use consumer price index (CPI) to deflate and then use regression analysis to forecast the future dynamics of material prices[4]. It is common knowledge that the purchasing power of a dollar in 1850 is significantly higher than that of a dollar today. The extent of changes in the purchasing power of a dollar between a given year and the base year is measured by price indices. The CPI is the most commonly used price index to quantify the purchasing power of a dollar in a given year relative to the given base-year, which is based on the values of a basket of items a representative consumer would buy (e.g., foods, housing, transport entertainment etc.) in the given year and the base-year. Understanding how the price of the metal in question increases or decreases in relation to the price of a standard basket of goods will give better insight than looking at the nominal price in isolation. Therefore, we will use the CPI, which is released by the United States Department of Labor[5], to remove the effect of inflation as presented in Eq. 10 below.

$$\text{RealValue}_{Year\_j} = \text{NominalValue}_{Year\_q} \cdot \frac{\text{CPI}_{Year\_j}}{\text{CPI}_{Year\_q}} \tag{10}$$

In the Low scenarios, we predict the long-term changes in metal prices based on the regression of logged prices on logged demand quantity (Single-Factor Learning Curve)[5].

Note S3. Impacts of critical material price surges on battery cost

Since the critical materials are used in batteries, the change of battery cost should be clarified first, and then the influence of critical material prices on EV cost can be discussed. We select four major cathode materials, lithium (Li), cobalt (Co), nickel (Ni), and manganese (Mn), to approximately represent the material cost of LIBs. The assumption that four major cathode materials represent the whole cost of battery neglects the cost of "other" cathode active materials, including aluminum in NCA cathodes and iron in LFP cathodes. The reason is that each of them accounts for less than 2% of the cost of battery costs and their supply is much less constrained in comparison with the four critical materials[6-8].

The overall cost of a LIB and a PEM fuel cell was estimated by using economic modeling, which reflects the battery cost fluctuation caused by material price surges (Eq. 11)[9]:

$$Cj = [\sum_i (Mi \times Pi)] \times [\frac{1}{Rj}] \tag{11}$$

where for *j* = fuel cell, *i* = Pt, for *j* = LIBs, *i* = Li, Co, Ni, Mn; C_j is the total battery cost of different technologies; M_i is the material intensity; P_i is the material costs; and R_j is a ratio of the critical material costs to the total battery cost.

Note S4. The impact of critical material price changes on vehicle cost

The final step is to calculate the share of the battery cost increment driven by increased material price in the total cost of a vehicle. This share characterizes the material price surge risk associated with low-carbon transport technologies.

$$Percentage\ of\ vehicle\ cost\ increase\ (\%) = \frac{The\ increase\ of\ battery\ costs(\$/veh)}{Vehicle\ cost\ under\ BLS\ scenario(\$/veh)} \times 100\% \tag{12}$$

Note S5. The vehicle stock and sale calculation

Since the GCAM does not count the number of vehicles explicitly, a conversion of transportation service demand into the number of vehicles is required. The vehicle stock can be calculated using the following equation:

$$Veh = D \times L^{-1} \times VKT^{-1} \tag{13}$$

where *Veh* stands for the vehicle stock; *D* is the transportation demand (passenger-km or tonne-km); *L* is the load factor (persons or tonnes) per vehicle; *VKT* is the vehicle travelled kilometer (km/vehicle).

We then adopt a stock-driven dynamic material-flow-analysis (MFA) model to estimate the inflow (sale) and outflow (decommissioning) of vehicles[10, 11].

$$\text{outflow}_{EV}(t_n) = \sum_{t_0}^{t_{n-1}} (\text{inflow}_{EV}(t_i) \cdot [\text{survival}(t_{i-1} - t_0) - \text{survival}(t_i - t_0)] \tag{14}$$

Where the outflow in t_n ($\text{outflow}_{EV}(t_n)$) is the sum of decommissioning of past inflow vintages in $t_i \in (t_0, t_{n-1})$. Survival(Δt) is the complementary cumulative distribution function of the normal distribution[10]. In this study, the average lifetime is assumed to be 10 years, 13 years, and 15 years for LDV-4W, bus, and truck, respectively. According to the principle of conservation of mass, the inflow ($\text{inflow}_{EV}(t_n)$) must equal a combination of the changes in stock ($\text{stock}_{EV}(t_n) - \text{stock}_{EV}(t_{n-1})$) and all outflows during this period:

$$\text{inflow}_{EV}(t_n) = \text{stock}_{EV}(t_n) - \text{stock}_{EV}(t_{n-1}) + \text{outflow}_{EV}(t_n) \tag{15}$$

Note S6. The effects of second use and battery lifetime changes on material recycling

Closed-loop recycling of battery materials is an important source of future battery material supply[11], however, the changes in battery operation lifetime will have a significant impact on material recycling. Therefore, we couple the lifetime distribution delay forecasting model with the material flow analysis to analyze the recycling potential of battery materials by considering both direct and indirect battery returns from first use and second use[12]. We assume that the materials obtained by battery manufacturers through recycling are not affected by material price fluctuations on the international market, that is, only the primary demand for materials is affected by the surging material prices.

The lifecycle stages of an EV battery and the sources of waste batteries entering the recycling market are illustrated in Fig. S4. EV waste batteries entering the recycling market include direct and indirect sources. Indirect sources include decommissioned batteries after secondary applications (such as energy storage systems) and batteries replaced by early failures (replaced batteries can only be recycled). Batteries that do not belong to the early failure and cannot be echelon utilized and can only be recycled belong to the direct source. Here, early failure refers to a failure that occurs during the warranty period of an EV (8 years),

some of the battery faults (r (s)) can be repaired, reused, or remanufactured again for use in EVs, and other part of faulty batteries (1 - r (s)) can only be replaced, and the replaced batteries are recycled. The early failure remanufactured rate is assumed to be 70% in this research[14].

The amounts of replacements, $QR(w)$ at year w for batteries sold in year s is calculated using Eq. (16).

$$QR(w) = \sum_{s=w-8}^{s=w-1} QP(s) \times D_{EV}(s, w) \times (1 - r(s)) \quad (16)$$

Where $QP(s)$ is the total number of EVs put on the market in year s in the BLS scenario; $D_{EV}(s, w)$ is the probability of product failure in year w of a battery that started its use stage in year s; The average lifetime of the use stage is set to be 11 years, and the standard deviation is set as 1.8.

The direct waste batteries that fail outside the warranty period and directly flow onto the recycling market in year w ($DW(w)$) can be estimated with the following equation:

$$DW(w) = \sum_{s=1}^{s=w-9} (QP(s) + QR(s)) \times D_{EV}(s, w) \times S(w) \quad (17)$$

Where $S(w)$ is the share of recycling EV battery on EoL markets, which will be reduced from 90% in 2019 to 50% in 2030 and stay stable afterwards.

The amount of waste EV batteries flowing into the B2U application (Q_{B2U}) can be calculated by eq. (18):

$$Q_{B2U} = \sum_{s=1}^{s=w-9} (QP(s) + QR(s)) \times D_{EV}(s, w) \times (1 - S(w)) \quad (18)$$

Where 1-$S(w)$ is the share of second use EV battery on EoL markets.

The composition of retired EV batteries from B2U applications $IW_{B2U}(w)$, is formulated in Eq. (19).

$$IW_{B2U}(w) = \sum_{s=1}^{s=w-1} Q_{B2U}(s) \times D_{B2U}(s, w) \quad (19)$$

Where $D_{B2U}(s, w)$ is the probability of product failure in year w of a battery that started its use stage in year s. The average lifetime of the lifetime of EV batteries in B2U is set to be 5 years, and the standard deviation is set as 2.6.

Thus, the total waste stream returning to recycling in year w ($TW(w)$), termed as the sum of direct recycled batteries (DW), waste batteries from second use applications (IW_{B2U}), and replacement EV batteries (QR), is given by Eq. (20).

$$TW(w) = DW(w) + IW_{B2U}(w) + QR(w) \quad (20)$$

The amount of recycling material in year w ($FC_{recycling}(w)$) can be estimated with Eq. (21):

$$FC_{recycling}(w) = TW(w) \times Cap_{EV}(s) \times C_i(s) \quad (21)$$

Where $Cap_{EV}(s)$ is their average capacity in kWh in year *s*, which is 35 kWh for LDV-4W, 70 kWh for bus, and 106 kWh for truck; $C_i(s)$ is material intensity of material *i* (kg/kWh) in EV batteries sold in year *s*, which is summarized in Table S2.

Supplementary Tables

ST1 Technology economic assumptions

Table S1. Shared Socioeconomic Pathway 1 (SSP1) for socioeconomic assumptions.

Year	Population (thous)	GDP (Million1990US$)
2020	1379410	9076750
2025	1379360	12699900
2030	1368370	16797900
2035	1348130	20926400
2040	1318370	24612200
2045	1280030	27701200
2050	1234330	29916900
2055	1182920	31226000
2060	1127190	31972400

Table S2. Values used for each of the case study clean road transportation technology's economic calculations.

Battery (*j*)	Material contribution to battery cost (R_j): [13-15]	Battery component	Element (*i*)	Price, $/kg (*P*)	Average material intensity, kg/kWh (*M*)	Sources (*P* and *M*)
Li-ion battery (NMC111)	0.196	NMC111 cathode active materials	Li	6.75	0.118	8,16,17
			Co	34.19	0.313	8,16,18
			Mn	2.04	0.292	8,16,19
			Ni	13.91	0.312	8,16,18
Li-ion battery (NMC622)	0.157	NMC622 cathode active materials	Li	6.75	0.100	8,16,17
			Co	34.19	0.170	8,16,18
			Mn	2.04	0.159	8,16,19
			Ni	13.91	0.508	8,16,18
Li-ion battery (NMC811)	0.133	NMC811 cathode active materials	Li	6.75	0.090	8,16,17
			Co	34.19	0.076	8,16,18
			Mn	2.04	0.071	8,16,19
			Ni	13.91	0.608	8,16,18
Li-ion battery (NMC9.5.5)	0.122	NMC9.5.5 cathode active materials	Li	6.75	0.090	8,16,17
			Co	34.19	0.037	8,16,18
			Mn	2.04	0.035	8,16,19
			Ni	13.91	0.683	8,16,18
Li-ion battery (NCA)	0.194	NCA cathode active materials	Li	6.75	0.106	8,16,17
			Co	34.19	0.117	8,16,18
			Mn	2.04	0.000	8,16,19
			Ni	13.91	0.618	8,16,18
Li-ion battery (LFP)	0.114	LFP cathode active materials	Li	6.75	0.087	8,16,17
			Co	34.19	0.000	8,16,18
			Mn	2.04	0.000	8,16,19
			Ni	13.91	0.000	8,16,18
Li-ion battery (LMO)	0.103	LMO cathode active materials	Li	6.75	0.097	8,16,17
			Co	34.19	0.000	8,16,18
			Mn	2.04	0.103	8,16,19
			Ni	13.91	0.000	8,16,18
**Battery (*j*)**	**Battery cost, $/ kW ($C_j$) Sources: [20-23]**	**Battery component**	**Element (*i*)**	**Price, $/kg (*P*)**	**Average material intensity, kg/kW (*M*)**	**Sources (*P* and *M*)**
PEM fuel cell system	55	Catalyst	Pt	26715.6	0.0002	18,24-26

ST2 Road transportation sector technology assumptions

Table S3. Road transport load factor (pers or tonnes/veh) in future scenarios from 2020–2060.

Vehicle size	2020	2025	2030	2035	2040	2045	2050	2055	2060
Compact Car	2.33	2.28	2.22	2.17	2.11	2.06	2	2	2
Large Car and SUV	2.50	2.50	2.50	2.50	2.50	2.50	2.50	2.50	2.50
Mini Car	2.33	2.28	2.22	2.17	2.11	2.06	2	2	2
Subcompact Car	2.33	2.28	2.22	2.17	2.11	2.06	2	2	2
Light Bus	20	20	20	20	20	20	20	20	20
Heavy Bus	40	40	40	40	40	40	40	40	40
Light Truck	0.52	0.52	0.52	0.52	0.52	0.52	0.52	0.52	0.52
Medium Truck	4.50	4.50	4.50	4.50	4.50	4.50	4.50	4.50	4.50
Heavy Truck	7.45	7.45	7.45	7.45	7.45	7.45	7.45	7.45	7.45

Table S4. Road transport vehicle traveled kilometer (VKT (km)) in future scenarios from 2020–2060[27,28].

Vehicle size	2020	2025	2030	2035	2040	2045	2050	2055	2060
Compact Car	14796	14796	14796	14796	14796	14796	14796	14796	14796
Large Car and SUV	14924	14924	14924	14924	14924	14924	14924	14924	14924
Mini Car	11466	11466	11466	11466	11466	11466	11466	11466	11466
Subcompact Car	17340	17340	17340	17340	17340	17340	17340	17340	17340
Light Bus	129800	131900	134000	136100	138200	140300	142400	142400	142400
Heavy Bus	129800	131900	134000	136100	138200	140300	142400	142400	142400
Light Truck	32750	34000	34750	35500	35500	35500	35500	35500	35500
Medium Truck	81159	83727	85295	86863	86863	86863	86863	86863	86863
Heavy Truck	86000	88700	90350	92000	92000	92000	92000	92000	92000

Table S5. Road transport vehicle cost (1990$/vehicle-km) in BLS scenario from 2020–2060.

Mode	Tech	2020	2025	2030	2035	2040	2045	2050	2055	2060
Compact Car	ICEV	0.2007	0.2049	0.2092	0.2135	0.2135	0.2135	0.2135	0.2135	0.2135
	BEV	0.2333	0.2151	0.1969	0.1786	0.1777	0.1767	0.1757	0.1754	0.1751
	FCEV	0.2416	0.2227	0.2037	0.1847	0.1840	0.1834	0.1827	0.1827	0.1827
	NGV	0.2291	0.2364	0.2437	0.2510	0.2511	0.2512	0.2513	0.2513	0.2513
	HEV	0.2230	0.2303	0.2376	0.2450	0.2450	0.2450	0.2450	0.2450	0.2450
Large Car and SUV	ICEV	0.3478	0.3549	0.3621	0.3693	0.3693	0.3693	0.3692	0.3693	0.3693
	BEV	0.3898	0.3596	0.3295	0.2993	0.2976	0.2959	0.2941	0.2937	0.2932
	FCEV	0.3775	0.3589	0.3403	0.3127	0.3081	0.3035	0.3079	0.3078	0.3077
	NGV	0.3849	0.3971	0.4093	0.4215	0.4216	0.4218	0.4219	0.4219	0.4219
	HEV	0.3712	0.3834	0.3956	0.4078	0.4078	0.4078	0.4078	0.4078	0.4078
Mini Car	ICEV	0.0724	0.0754	0.0785	0.0815	0.0815	0.0815	0.0815	0.0815	0.0815
	BEV	0.0963	0.0873	0.0783	0.0693	0.0689	0.0686	0.0682	0.0681	0.0680
	FCEV	0.1088	0.0990	0.0891	0.0793	0.0790	0.0788	0.0786	0.0786	0.0786
	NGV	0.0769	0.0800	0.0830	0.0860	0.0860	0.0860	0.0860	0.0860	0.0860
	HEV	0.0786	0.0817	0.0847	0.0877	0.0877	0.0877	0.0877	0.0877	0.0877
Subcompact Car	ICEV	0.1099	0.1142	0.1185	0.1229	0.1229	0.1229	0.1229	0.1229	0.1229
	BEV	0.1563	0.1399	0.1253	0.1072	0.1066	0.1060	0.1054	0.1052	0.1050
	FCEV	0.1570	0.1439	0.1308	0.1177	0.1173	0.1169	0.1165	0.1165	0.1165
	NGV	0.1197	0.1240	0.1284	0.1327	0.1327	0.1327	0.1327	0.1327	0.1327
	HEV	0.1223	0.1266	0.1309	0.1353	0.1353	0.1353	0.1353	0.1353	0.1353
Light Bus	ICEV	0.7107	0.7107	0.7107	0.7107	0.7107	0.7107	0.7107	0.7107	0.7107
	BEV	0.9700	0.8300	0.6000	0.5900	0.5800	0.5700	0.5700	0.5700	0.5700
	FCEV	1.1500	0.9900	0.7100	0.5900	0.5200	0.4900	0.4900	0.4800	0.4800
	NGV	0.7669	0.7669	0.7669	0.7669	0.7669	0.7669	0.7669	0.7669	0.7669
Heavy Bus	ICEV	0.8275	0.8275	0.8275	0.8275	0.8275	0.8275	0.8275	0.8275	0.8275
	BEV	1.0800	0.9300	0.6700	0.6600	0.6500	0.6500	0.6300	0.6300	0.6300
	FCEV	1.2900	1.1100	0.8000	0.6600	0.5900	0.5500	0.4900	0.4800	0.4800
	NGV	0.8930	0.8930	0.8930	0.8930	0.8930	0.8930	0.8930	0.8930	0.8930
Light Truck	ICEV	0.2973	0.2973	0.2973	0.2973	0.2973	0.2973	0.2973	0.2973	0.2973
	BEV	0.5100	0.3600	0.3000	0.2500	0.2200	0.1900	0.1600	0.1600	0.1600
	FCEV	0.7100	0.4000	0.3200	0.2300	0.2000	0.1700	0.1400	0.1400	0.1400
	NGV	0.3831	0.3805	0.3779	0.3754	0.3727	0.3701	0.3675	0.3675	0.3675
Medium Truck	ICEV	0.3314	0.3314	0.3314	0.3314	0.3314	0.3314	0.3314	0.3314	0.3314
	BEV	0.5300	0.3700	0.3200	0.2600	0.2300	0.2000	0.1700	0.1700	0.1700
	FCEV	0.7400	0.4200	0.3300	0.2400	0.2100	0.1800	0.1500	0.1500	0.1500
	NGV	0.4273	0.4244	0.4215	0.4185	0.4156	0.4127	0.4098	0.4098	0.4098
Heavy Truck	ICEV	0.3466	0.3466	0.3466	0.3466	0.3466	0.3466	0.3466	0.3466	0.3466
	BEV	0.6100	0.4300	0.3700	0.3000	0.2600	0.2300	0.1900	0.1900	0.1900
	FCEV	0.8500	0.4800	0.3800	0.2800	0.2400	0.2100	0.1700	0.1700	0.1700
	NGV	0.4467	0.4437	0.4407	0.4376	0.4346	0.4316	0.4286	0.4286	0.4286

Table S6. Comparison to results from literature for the NMC111 LIB chemistry. Comparison shown for this study versus the Bloomberg New Energy Finance Group report[29].

100% Price Increase in Material Price	Change in NMC111 Battery Cost (BNEF [29])	Change in NMC111 Battery Cost (This Study)
Lithium	8%	5%
Cobalt	20%	12%
Nickel	3%	2%

Table S7. Comparison to results from literature for the EV development under material shortage constraint. Comparison shown for this study versus Liu et al.[30]

Scenario		EV stock in 2050 (million)	EV market share in 2050 (%)
Liu et al.	NMC	685.8	89.0
	LS	226.1	46.8
	HS	301.2	62.3
This study	BLS	489.8	70.7
	High	217.2	33.5
	Medium	394.7	60.9
	Low	463.5	68.8

Note: This study includes scenarios in which EVs are equipped with different types of LIBs, and results in this table in which EVs are equipped with NCM111 LIBs. *Abbreviations*: NMC, Non-metal-constraints; LS, Low metal supply; HS, High metal supply; BLS, Absence of surge in material prices; High, High level of surge in material prices; Medium, Medium surge in material prices; Low, Low surge in material prices.

1.3 Literature review on future vehicle flow and stock studies

Table S8. Literature review on vehicle flow and stock projection.

Author	Method	Key results
Milovanoff et al. (2020)[31]	GCAM:<br>The demand for passenger transportation services depends on per-capita GDP, the aggregated service price across all modes, the population, and income and price elasticities. Then, the market shares by mode and technology are determined using a logit formulation based on the cost of transport service and other cost parameters.	Current US policies are insufficient to remain within a sectoral CO_2 emission budget for light-duty vehicles, consistent with preventing more than 2 °C global warming, creating a mitigation gap of up to 19 $GtCO_2$ (28% of the projected 2015–2050 light-duty vehicle fleet emissions). Closing the mitigation gap solely with EVs would require more than 350 million on-road EVs (90% of the fleet), half of national electricity demand and excessive amounts of critical materials to be deployed in 2050.
McCollum et al. (2018)[32]	Six global energy economy modelling frameworks were employed in this study: GEM-E3T-ICCS, IMACLIM-R, IMAGE, MESSAGE-Transport, TIAM-UCL and WITCH.<br>(1) GEM-E3T-ICCS: The stock of vehicles by transport sector and the cars, represented as durable goods in the modelling of behavior of households, change over time as a result of mobility and scrappage. The choice of between vehicle technologies depends on relative costs, which include purchasing cost, running costs and cost factors reflecting uncertainty factors.<br>(2) IMACLIM-R: The service demand is determined by demography and labor productivity growth, the maximum potentials of technologies, the learning rates decreasing the cost of technologies, fossil fuel reserves, the parameters of the functions representing energy-efficiency in end-uses, and the parameters of the functions representing energy-demand behaviors and life-styles.<br>(3) IMAGE: The service demand is determined by GDP and population projections.<br>(4) MESSAGE-Transport: Future demand for passenger travel in the various modes is projected on a passenger-kilometer (pkm) basis as a function of per-capita GDP.<br>(5) TIAM-UCL: The service demands projected are calculated from a set of exogenously defined drivers (e.g., GDP, population, number of households); the	A diverse set of measures targeting vehicle buyers is necessary to drive widespread adoption of clean technologies. Carbon pricing alone is insufficient to bring low-carbon vehicles to the mass market, though it may have a supporting role in ensuring a decarbonized energy supply.

	demands respond to prices.<br>(6) WITCH: Transport demand is explicitly calculated based on GDP and population projections.	
Isik et al. (2021)[33]	COMET model:<br>Transport demands are derived by gross domestic product, population, etc.	The electrification of light-duty vehicles at earlier periods is essential for deeper reductions in air emissions. When further combined with energy efficiency improvements, these actions contribute to CO_2 reductions under the scenarios of more CO_2-intense electricity.
Baars et al. (2021)[34]	Ricardo Sultan model:<br>Projections for future car sales are based on the average car ownership per 1000 inhabitants in 2017, multiplied by future population projections.	The rapid development of EVs will lead to widespread adoption of LIBs, which will require increased natural resources for the automotive industry. The expected rapid increase in batteries could result in new resource challenges and supply-chain risks.
Hao et al. (2019)[11]	Transport Impact Model (TIM):<br>(1) Private passenger vehicle growth model: Automotive growth is correlated with household income growth and vehicle price variation.<br>(2) Urban public transportation vehicle growth model: Automotive growth is correlated with urbanization and population growth.<br>(3) Economic utility vehicle growth model: Automotive growth is correlated with GDP growth.	A mass electrification of the heavy-duty segment on top of the light-duty segment would substantially increase the lithium demand and impose further strain on the global lithium supply.
Peng et al. (2018)[35]	China Provincial Road Transport Energy Demand and GHG Emissions Analysis (CPREG) model:<br>(1) Non-taxi passenger vehicle stocks are projected with the Gompertz function relating vehicle ownership to per-capita GDP.<br>(2) The stock of commercial buses is the product of the ownership and population.<br>(3) Freight vehicles stock is assumed to be correlated to the elasticity between vehicle stock and GDP.	China's vehicle stock will keep increasing to 543 million by 2050. The spatial distributions of future vehicle stock, energy demand and GHG emissions vary among provinces and show a generally downward trend from east to west.
Pan et al. (2018)[36]	GCAM-TU:<br>(1) Passenger demand is determined by income (per capita GDP), population, and aggregate service price.<br>(2) Freight demand trajectory is estimated based on population and GDP that is subject to price-induced demand response.	China's transportation sector might need significant changes beyond 2030 to decouple associated CO_2 emissions from GDP growths. Supporting national mitigation has more pronounced implications on freight than passenger transport services, and arouses a radical shift of

		transport fuels away from fossil-based liquids to clean alternatives.
Khanna et al. (2021)[37]	Demand Resource Energy Analysis Model (DREAM):<br>The future sales and the implied stock of heavy-duty trucks is estimated by a bottom-up stock turnover model.	Beginning to deploy battery electric and fuel-cell heavy-duty trucks (HDTs) as early as 2020 and 2035, respectively, could achieve significant and the largest CO_2 emissions reduction by 2050 with a decarbonized power sector.

Supplementary Figures

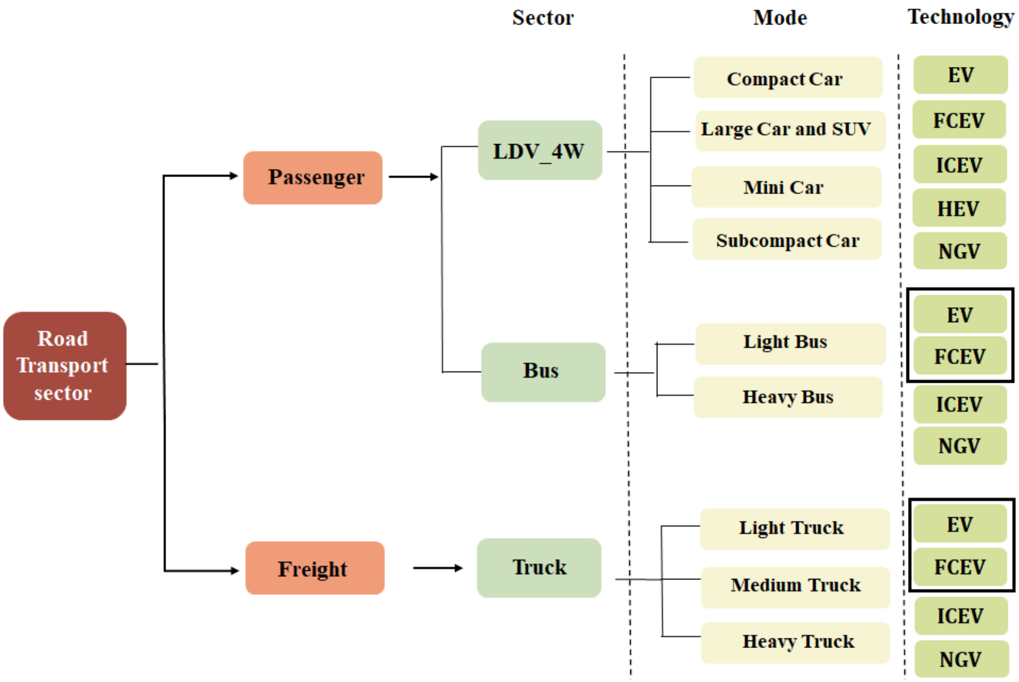

Figure S1. The structure of China's road transport sector. The technologies in the black box are those which GCAM v5.2 does not contain but we have added. *Abbreviations*: LDV-4W, light duty vehicle-4 wheels; EV, electric vehicle (electric vehicle refers to battery electric vehicle in this paper); FCEV, fuel cell vehicle; ICEV, internal combustion engine vehicle; HEV, hybrid electric vehicle; NGV, natural gas vehicle.

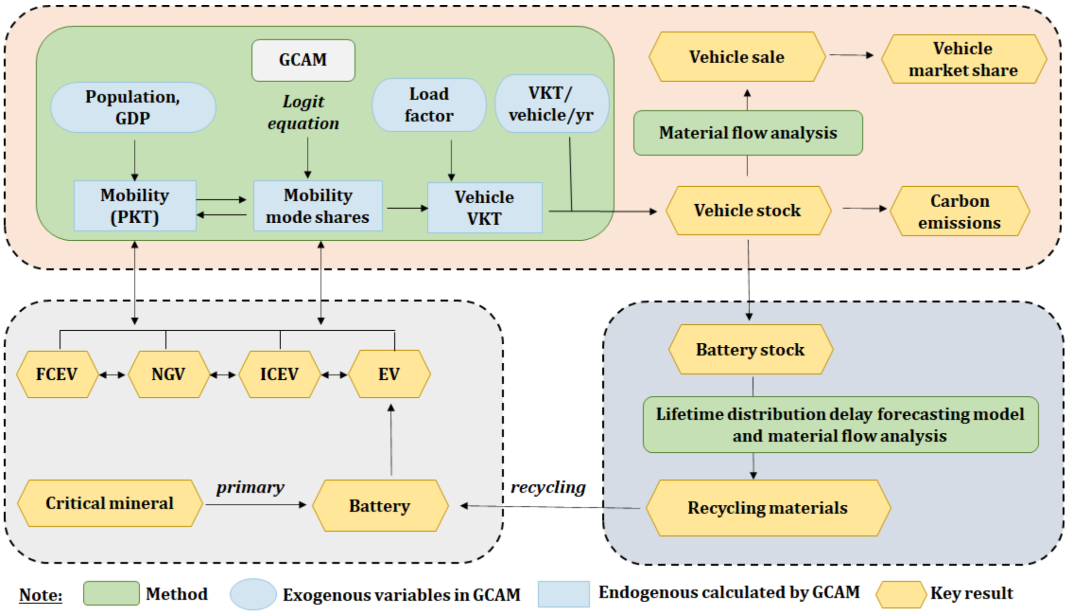

Figure S2. Schematic diagram of the analysis framework. VKT is vehicle kilometers travelled, PKT passenger kilometers travelled (PKT is related to VKT through the number of passengers per vehicle, which is sometimes called the occupancy rate); The abbreviations of EV, FCEV, ICEV, HEV, and NGV are the same as in Fig. S1.

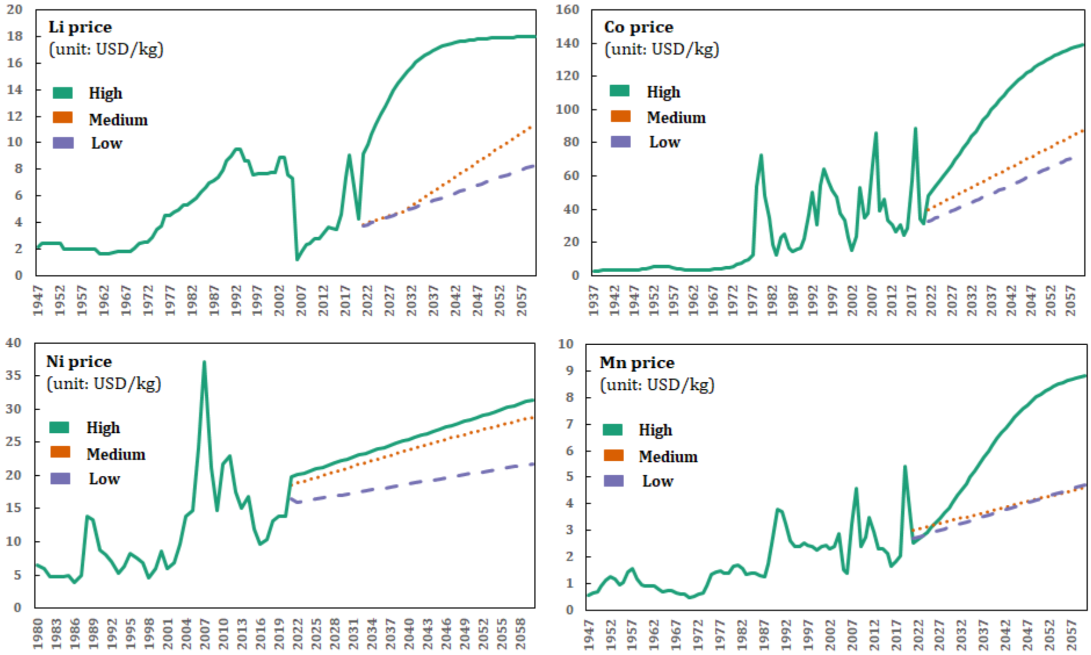

Figure S3. Historical and forecasted prices of critical materials. High scenario in which a rapid increase in critical material price affects EV costs; Medium scenario in which a steady increase in critical material price affects EV costs; Low scenario in which a slight increase in critical material price mainly affects EV costs during the middle and later periods of the forecast.

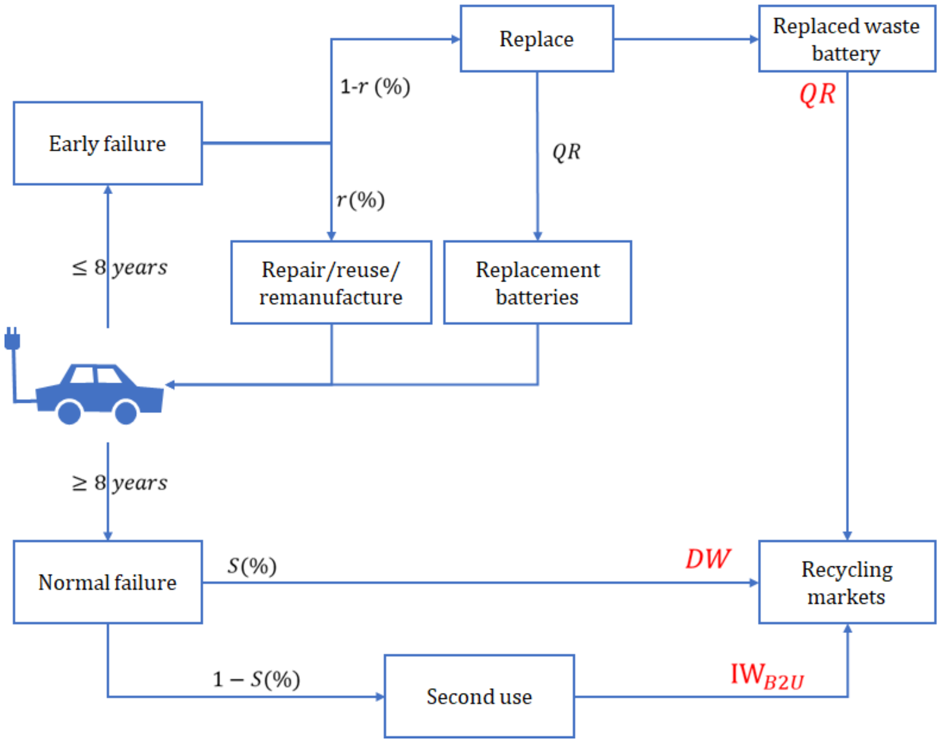

Figure S4. Sources of waste batteries entering the recycling market. *r* is the share of early failures of batteries during the warranty period that can be remanufactured; *S* is the share of recycling on EoL markets; *QR* is the amount of replacement EV batteries; *DW* is the amount of direct waste EV batteries; IW_{B2U} is the amount of waste EV batteries after second use applications.

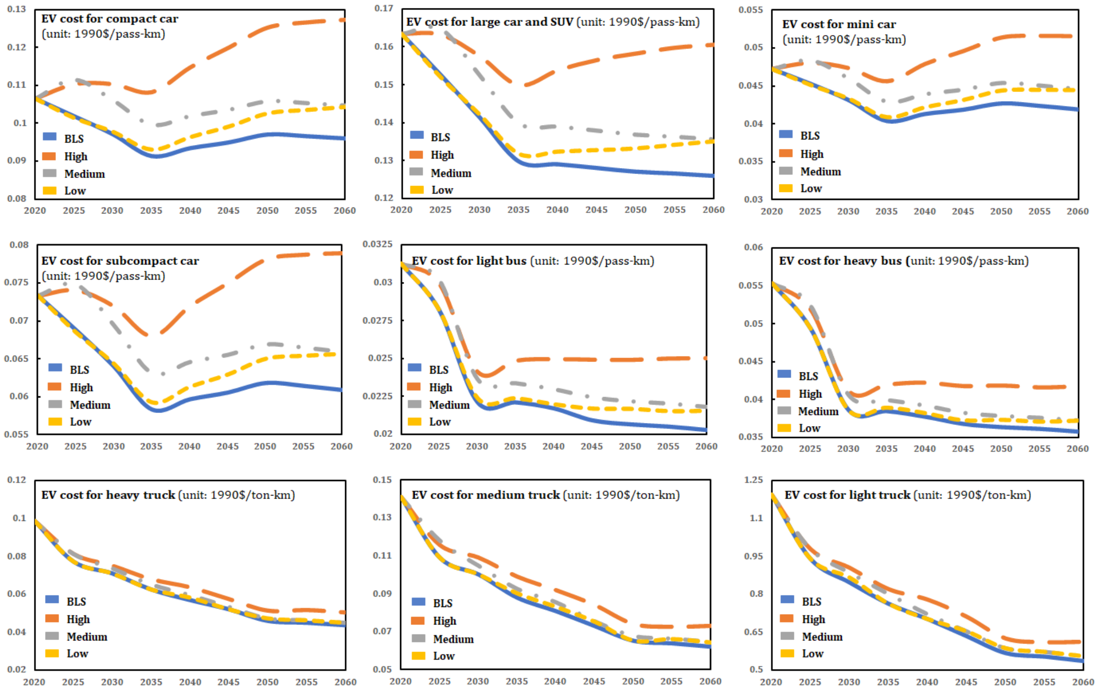

Figure S5. Cost evolution of EV equipped with NCM111 LIBs from 2020 to 2060. BLS refers to the base-line scenario in which the uptake pace of EVs will fulfil the requirement of the carbon neutrality target and the EV cost will fall rapidly in line with its historical and forecasted development trend in China as reported in the existing literature; The scenarios of High, Medium, and Low are the same as in Fig. S3; The abbreviation of EV is the same as in Fig. S1.

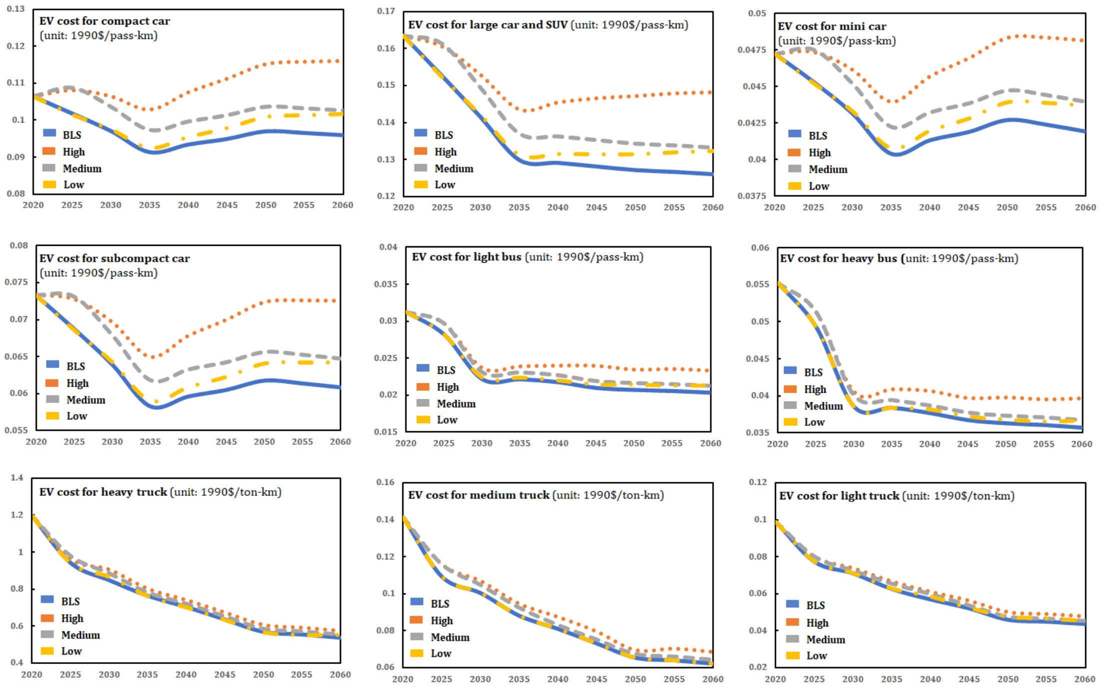

Figure S6. Cost evolution of EV equipped with NCM622 LIBs from 2020 to 2060. *Note:* The scenario of BLS is the same as in Fig. S5; The scenarios of High, Medium, and Low are the same as in Fig. S3; The abbreviation of EV is the same as in Fig. S1.

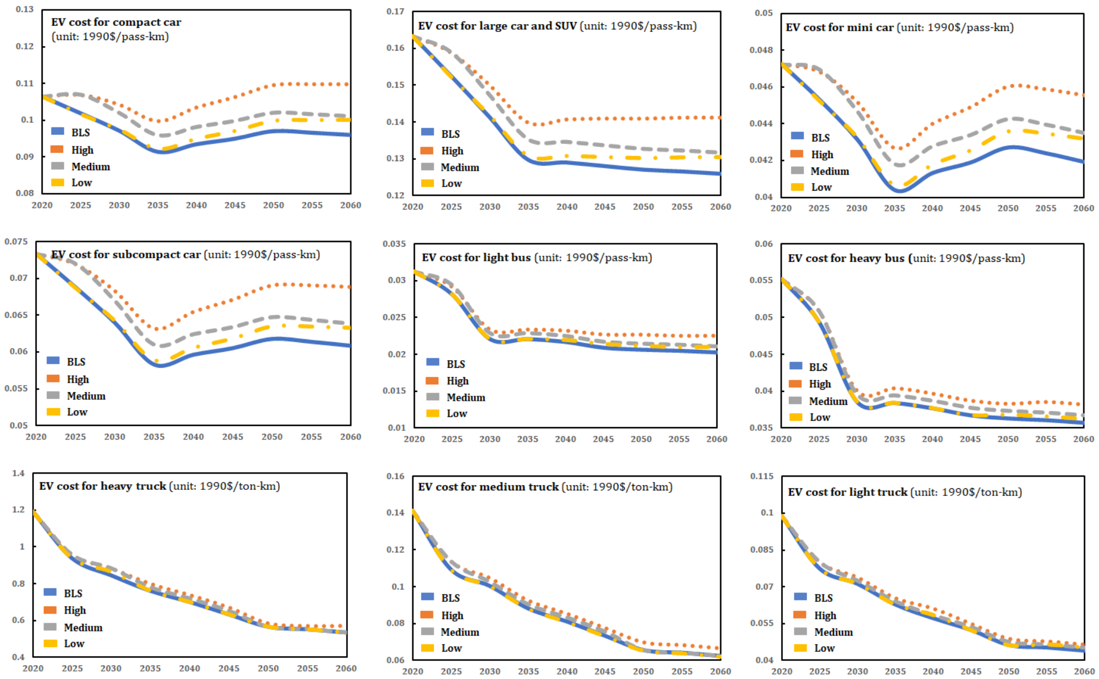

Figure S7. Cost evolution of EV equipped with NCM811 LIBs from 2020 to 2060. The scenario of BLS is the same as in Fig. S5; The scenarios of High, Medium, and Low are the same as in Fig. S3; The abbreviation of EV is the same as in Fig. S1.

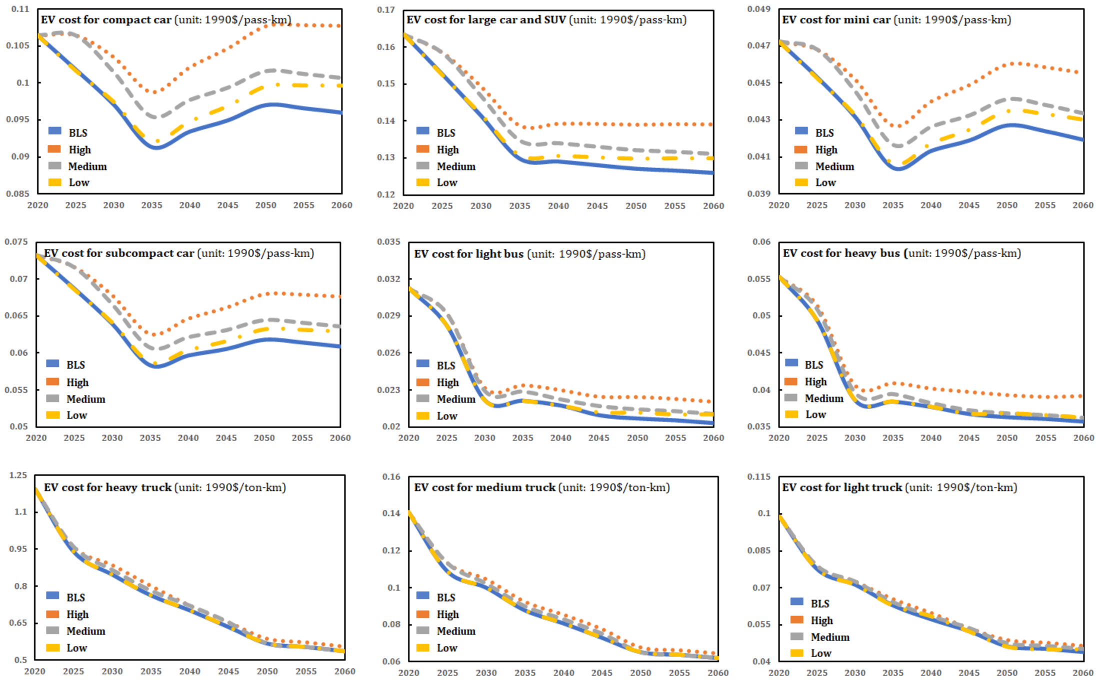

Figure S8. Cost evolution of EV equipped with NCM9.5.5 LIBs from 2020 to 2060. The scenario of BLS is the same as in Fig. S5; The scenarios of High, Medium, and Low are the same as in Fig. S3; The abbreviation of EV is the same as in Fig. S1.

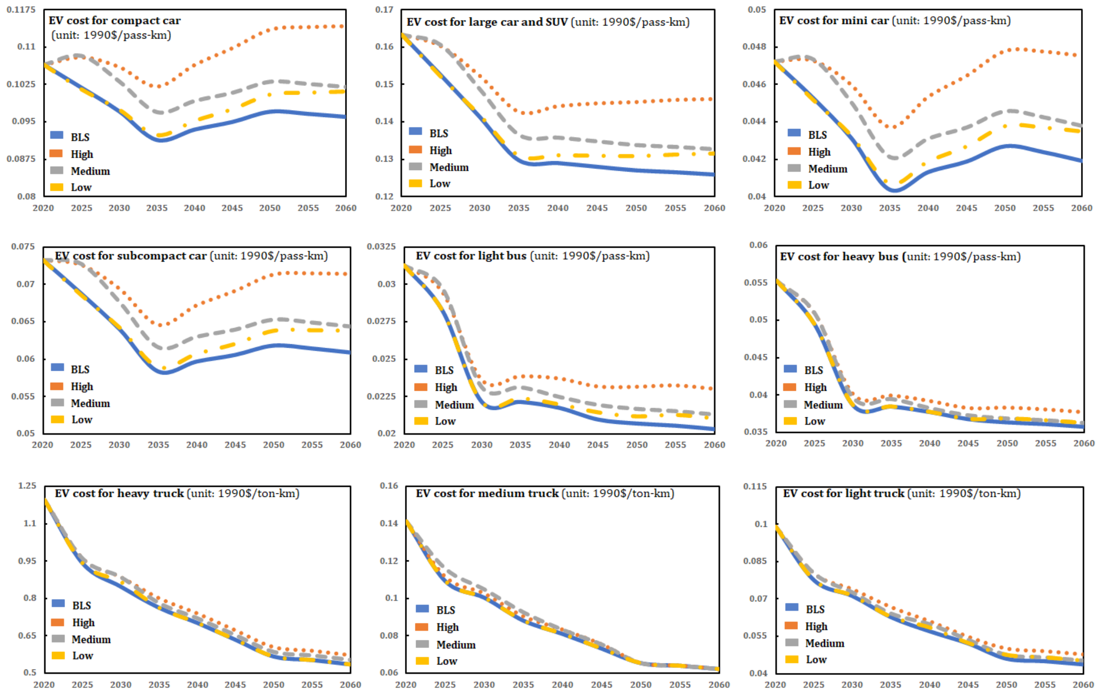

Figure S9. Cost evolution of EV equipped with NCA LIBs from 2020 to 2060. The scenario of BLS is the same as in Fig. S5; The scenarios of High, Medium, and Low are the same as in Fig. S3; The abbreviation of EV is the same as in Fig. S1.

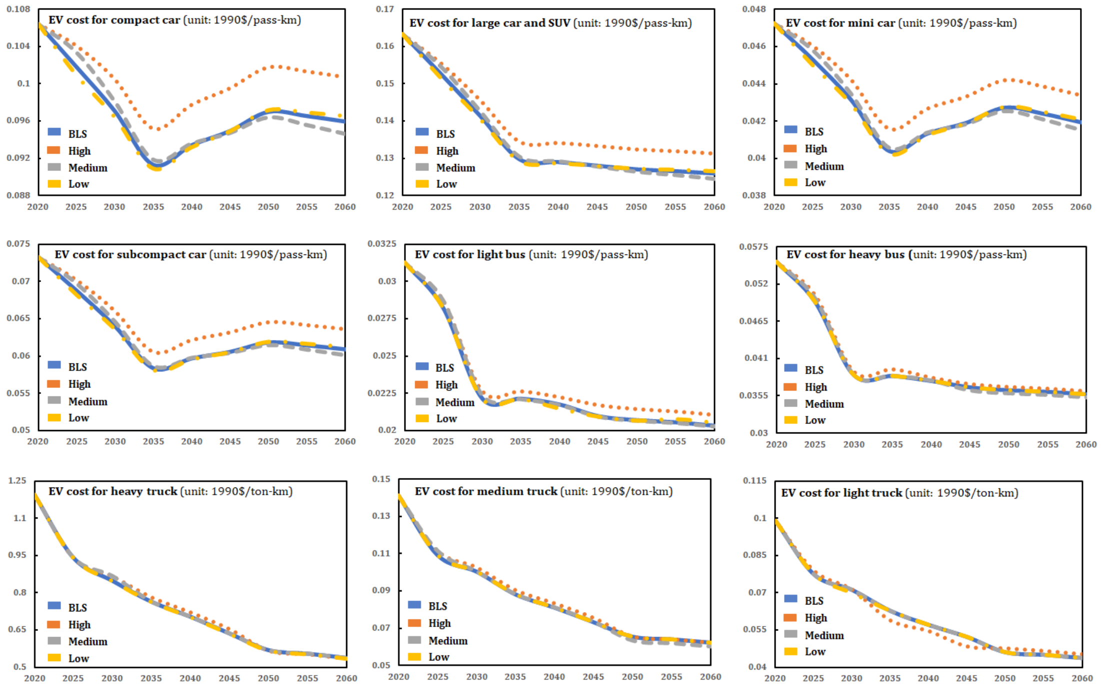

Figure S10. Cost evolution of EV equipped with LFP LIBs from 2020 to 2060. The scenario of BLS is the same as in Fig. S5; The scenarios of High, Medium, and Low are the same as in Fig. S3; The abbreviation of EV is the same as in Fig. S1.

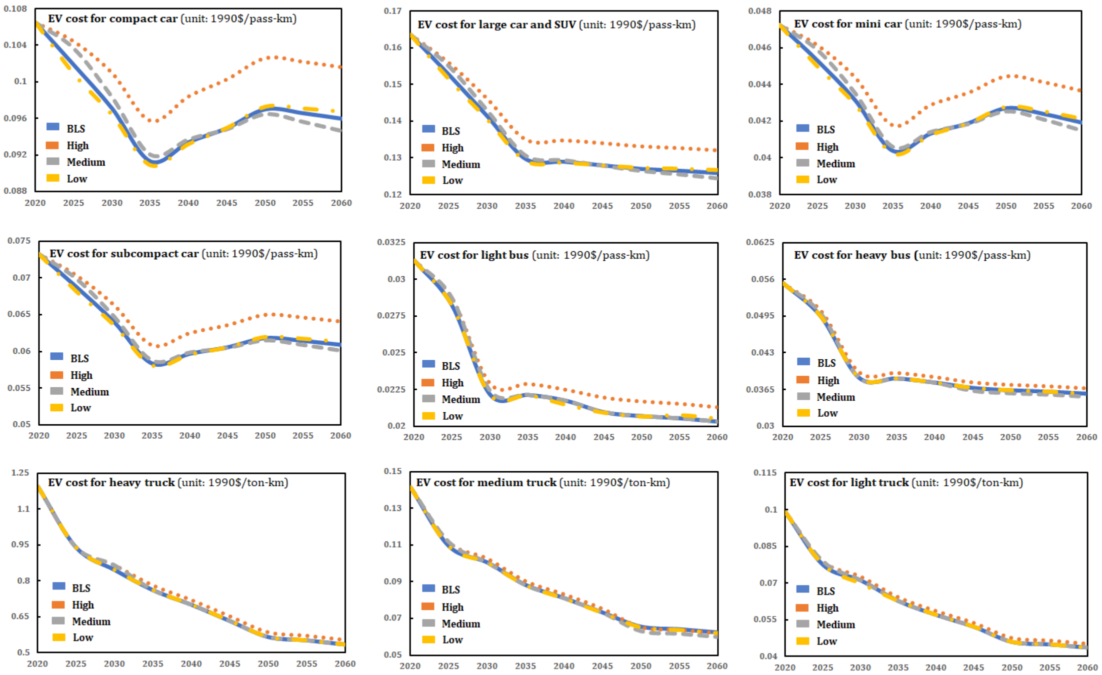

Figure S11. Cost evolution of EV equipped with LMO LIBs from 2020 to 2060. The scenario of BLS is the same as in Fig. S5; The scenarios of High, Medium, and Low are the same as in Fig. S3; The abbreviation of EV is the same as in Fig. S1.

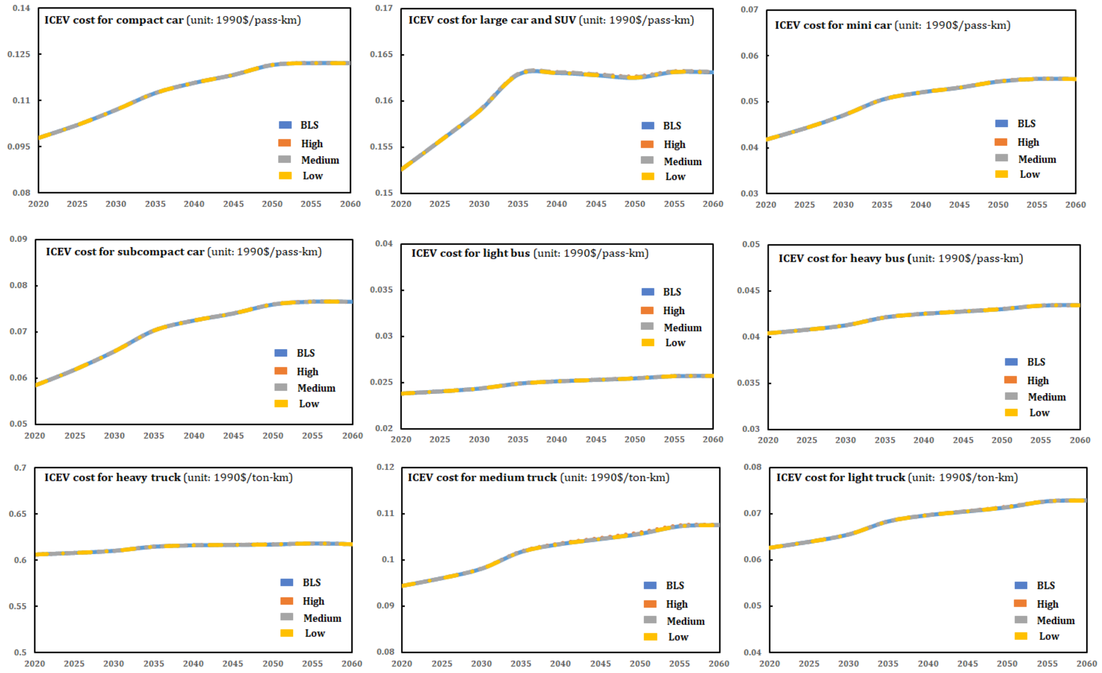

Figure S12. Cost evolution of ICEV from 2020 to 2060. The scenario of BLS is the same as in Fig. S5; The scenarios of High, Medium, and Low are the same as in Fig. S3; The abbreviation of ICEV is the same as in Fig. S1.

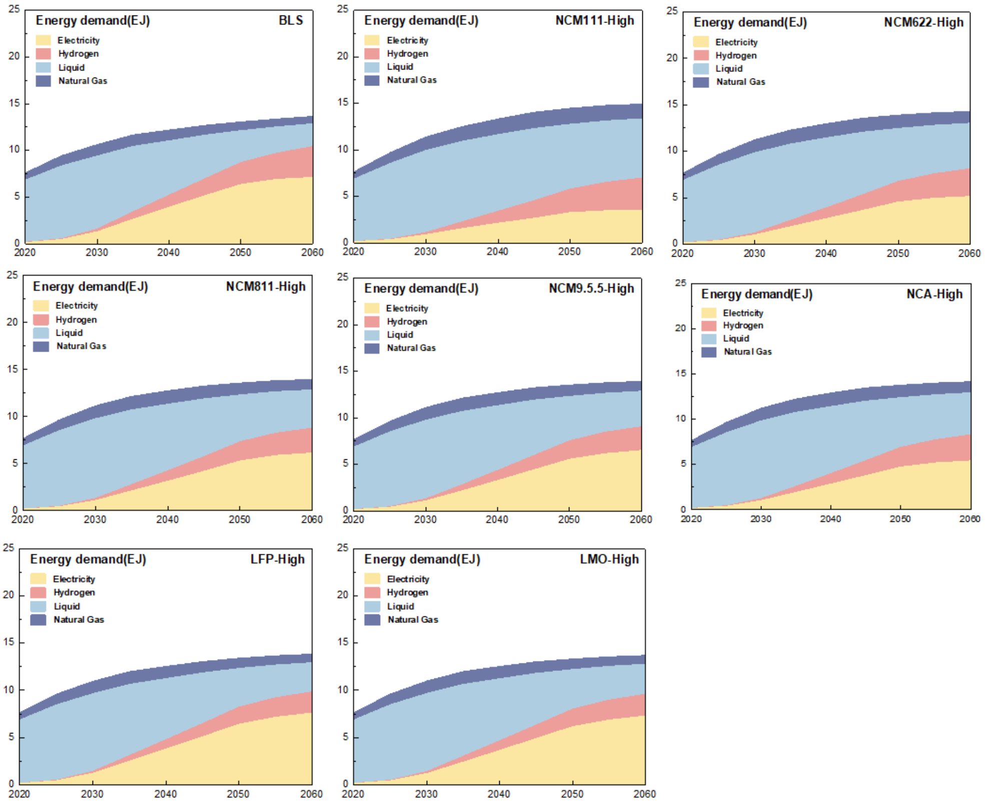

Figure S13. Energy demand by fuel in the High scenario from 2020 to 2060. The scenario of BLS is is the same as in Fig. S5; The scenario of High is the same as in Fig. S3.

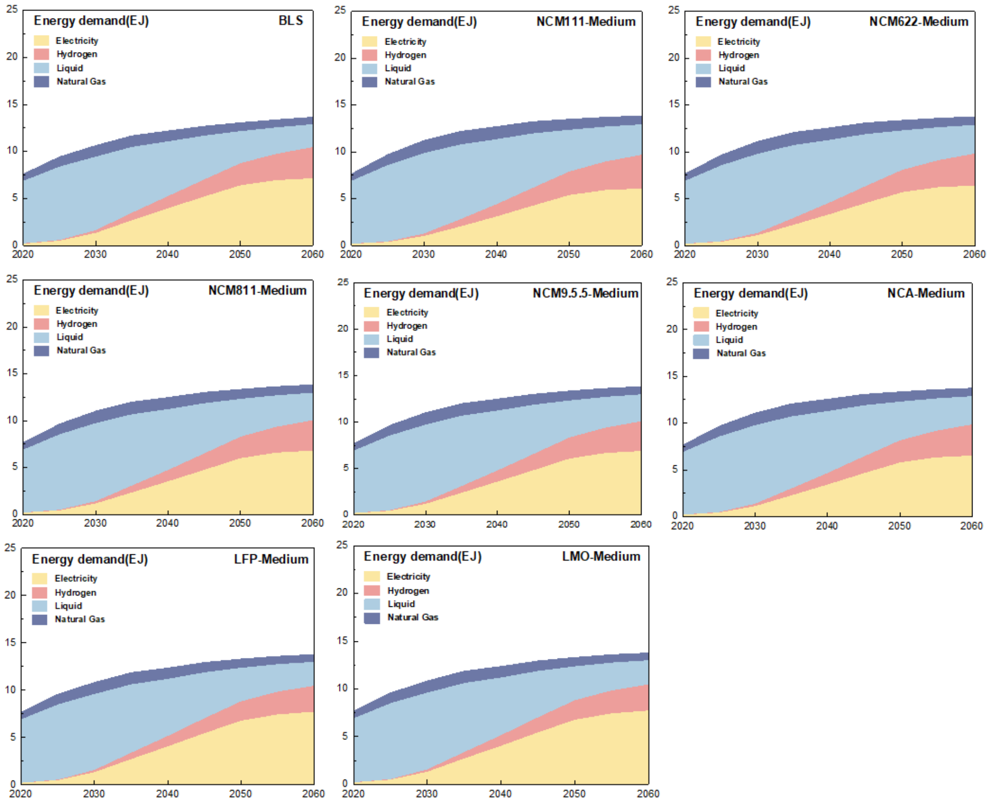

Figure S14. Energy demand by fuel in the Medium scenario from 2020 to 2060. The scenario of BLS is the same as in Fig. S5; The scenario of Medium is the same as in Fig. S3.

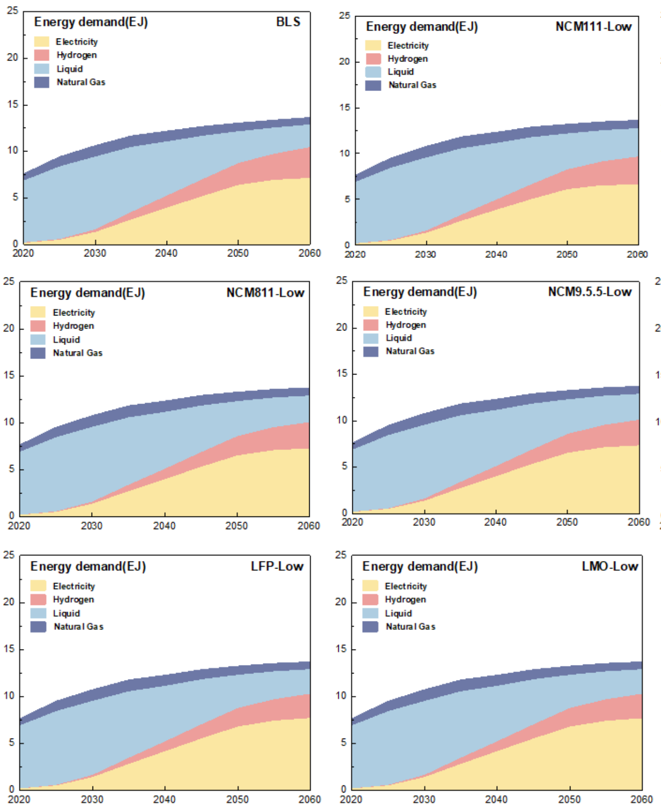

Figure S15. Energy demand by fuel in the Low scenario from 2020 to 2060. The scenario of BLS is the same as in Fig. S5; The scenario of Low is the same as in Fig. S3.

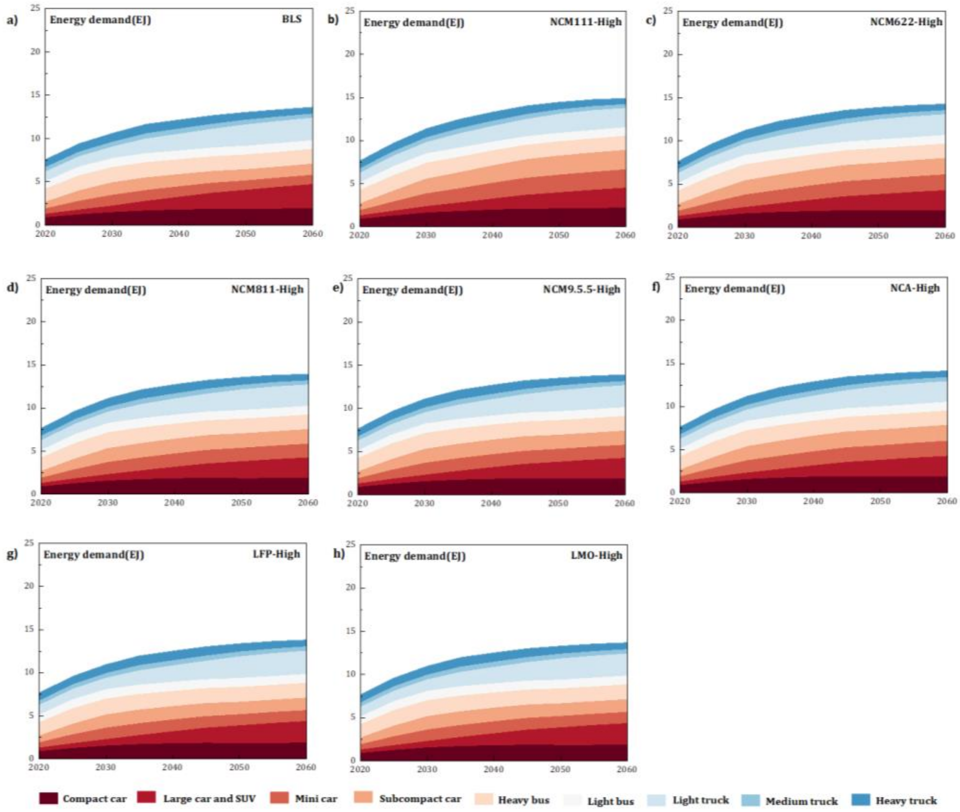

Figure S16. Energy demand by sector in the High scenarios from 2020 to 2060. The scenario of BLS is the same as in Fig. S5; The scenario of High is the same as in Fig. S3.

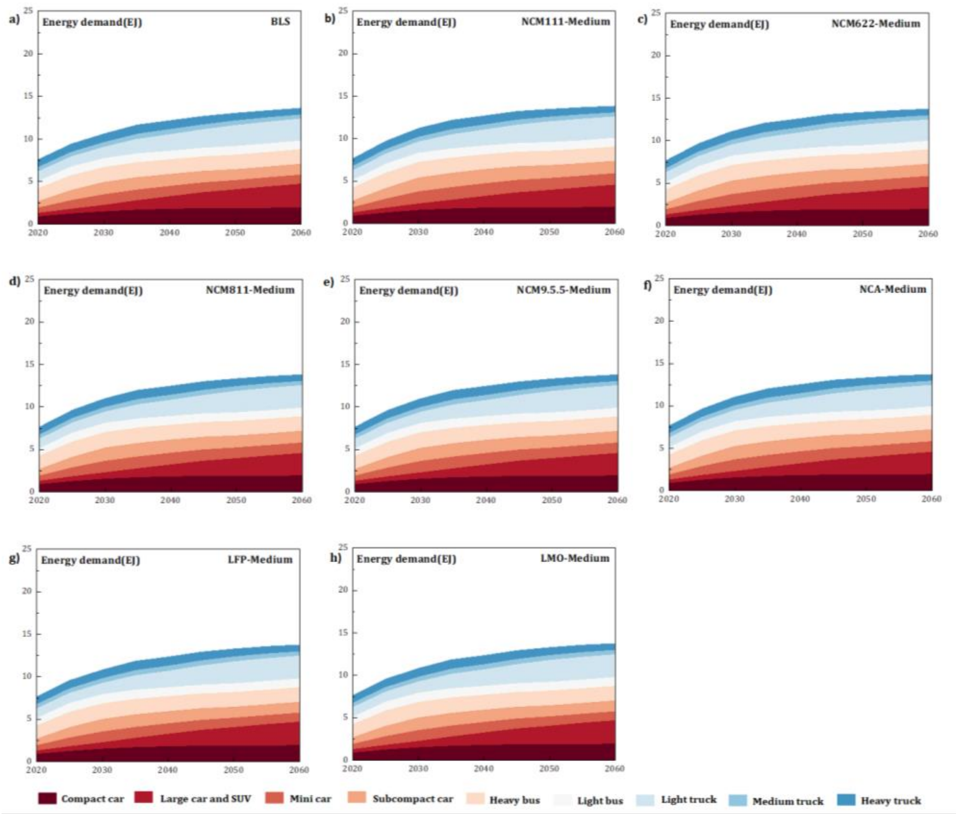

Figure S17. Energy demand by sector in the Medium scenarios from 2020 to 2060. The scenario of BLS is the same as in Fig. S5; The scenario of Medium is the same as in Fig. S3.

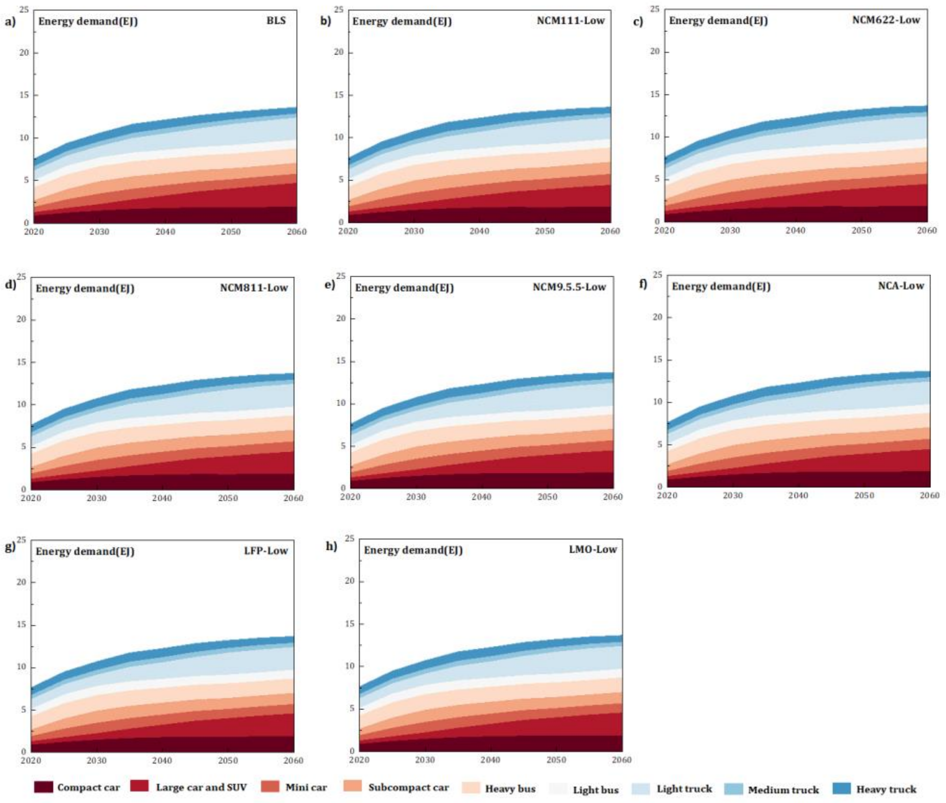

Figure S18. Energy demand by sector in the Low scenarios from 2020 to 2060. The scenario of BLS is the same as in Fig. S5; The scenario of Low is the same as in Fig. S3.

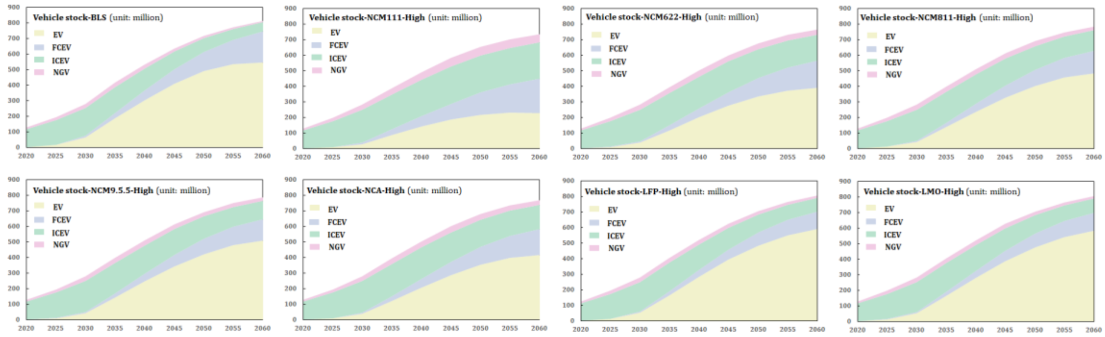

Figure S19. Vehicle stock in the High scenarios from 2020 to 2060. The abbreviations of EV, FCEV, ICEV, HEV, and NGV are the same as in Fig. S1; The scenario of BLS is the same as in Fig. S5; The scenario of High, is the same as in Fig. S3.

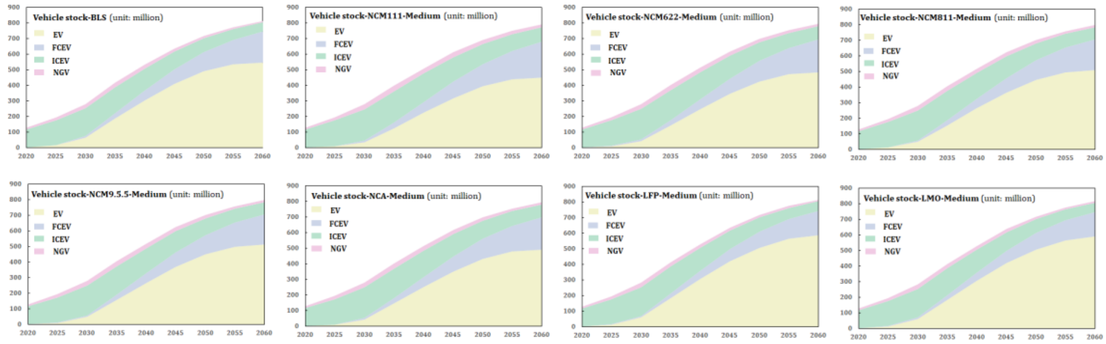

Figure S20. Vehicle stock in the Medium scenarios from 2020 to 2060. The abbreviations of EV, FCEV, ICEV, HEV, and NGV are the same as in Fig. S1; The scenario of BLS is is the same as in Fig. S5; The scenario of Medium is the same as in Fig. S3.

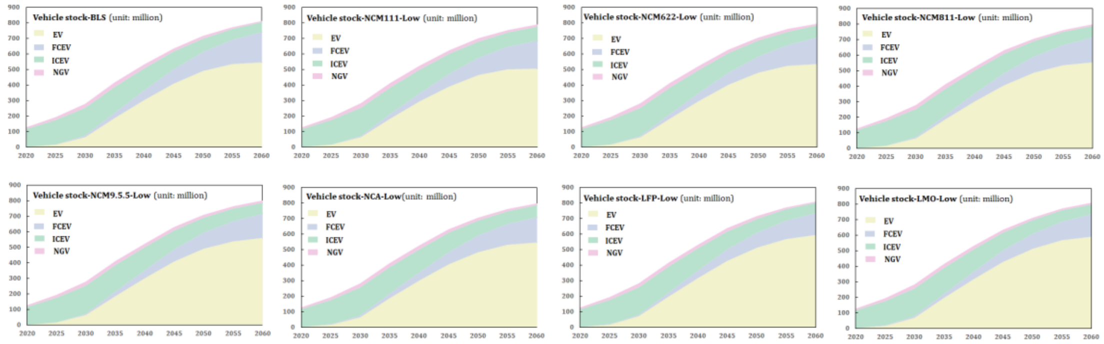

Figure S21. Vehicle stock in the Low scenarios from 2020 to 2060. The abbreviations of EV, FCEV, ICEV, HEV, and NGV are the same as in Fig. S1; The scenario of BLS is the same as in Fig. S5; The scenario of Low is the same as in Fig. S3.

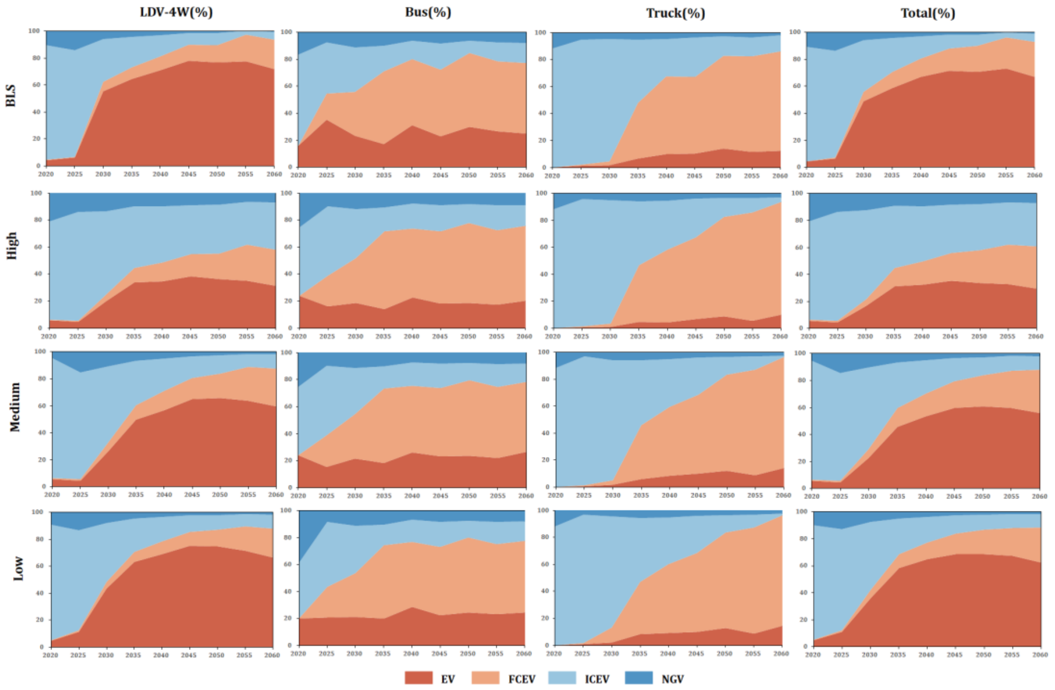

Figure S22. Vehicle penetration rate from 2020 to 2060 in which EVs are equipped with NCM111 LIBs. The abbreviations of EV, FCEV, ICEV, HEV, NGV, and LDV-4W are the same as in Fig. S1; The scenario of BLS is the same as in Fig. S5; The scenarios of High, Medium, and Low are the same as in Fig. S3.

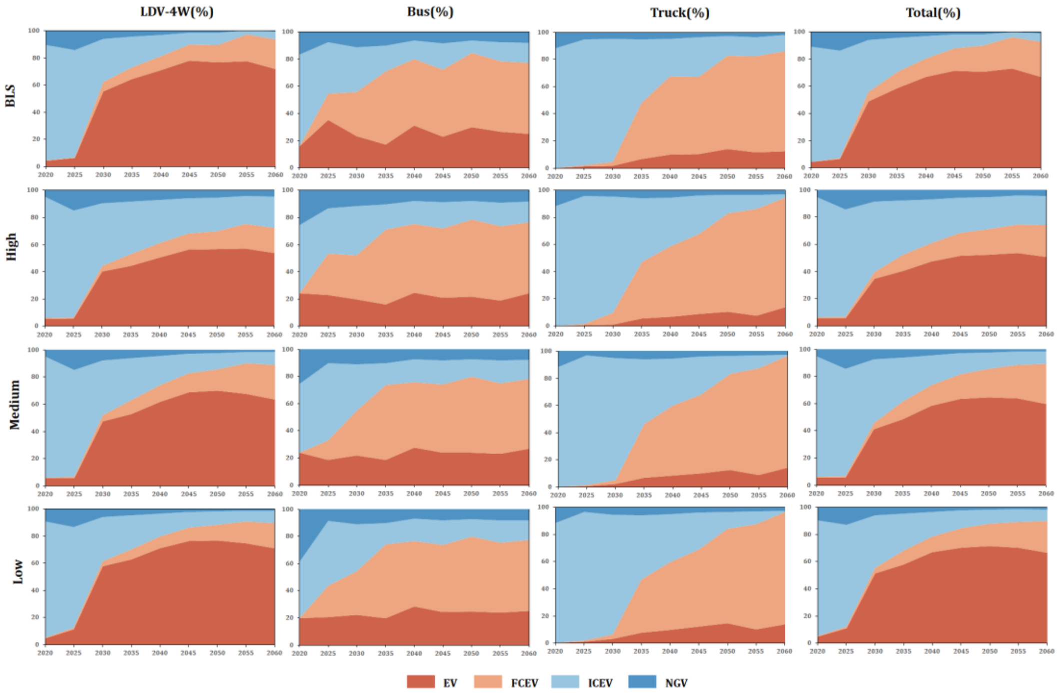

Figure S23. Vehicle penetration rate from 2020 to 2060 in which EVs are equipped with NCM622 LIBs. The abbreviations of EV, FCEV, ICEV, HEV, NGV, and LDV-4W are the same as in Fig. S1; The scenario of BLS is the same as in Fig. S5; The scenarios of High, Medium, and Low are the same as in Fig. S3.

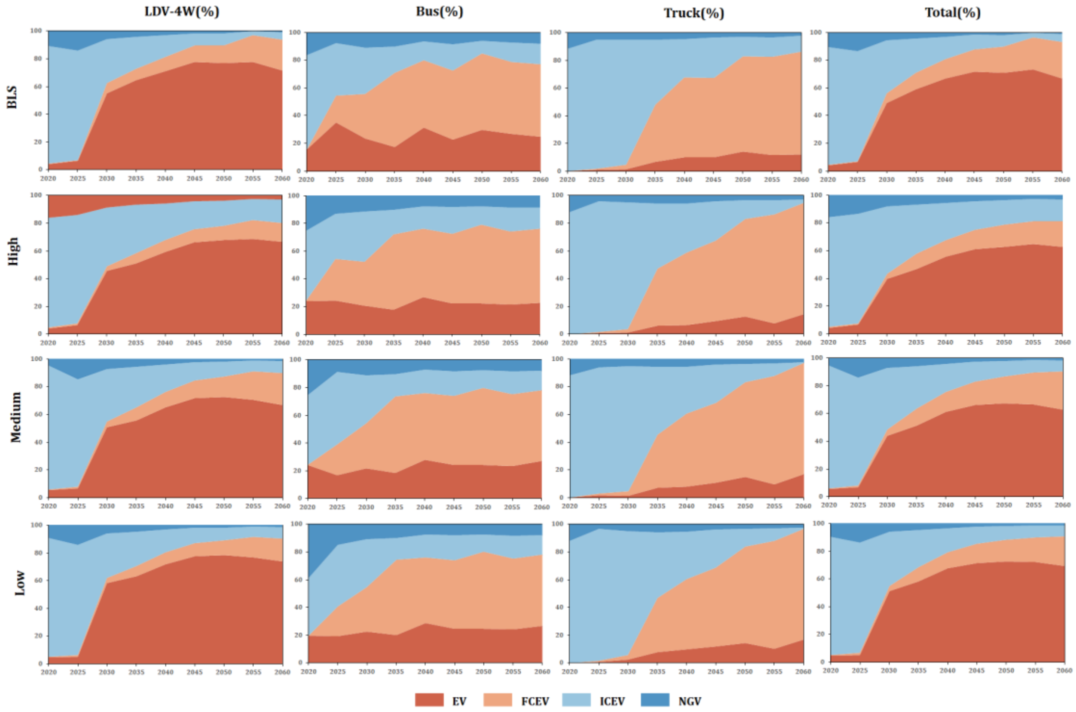

Figure S24. Vehicle penetration rate from 2020 to 2060 in which EVs are equipped with NCM811 LIBs. The abbreviations of EV, FCEV, ICEV, HEV, NGV, and LDV-4W are the same as in Fig. S1; The scenario of BLS is the same as in Fig. S5; The scenarios of High, Medium, and Low are the same as in Fig. S3.

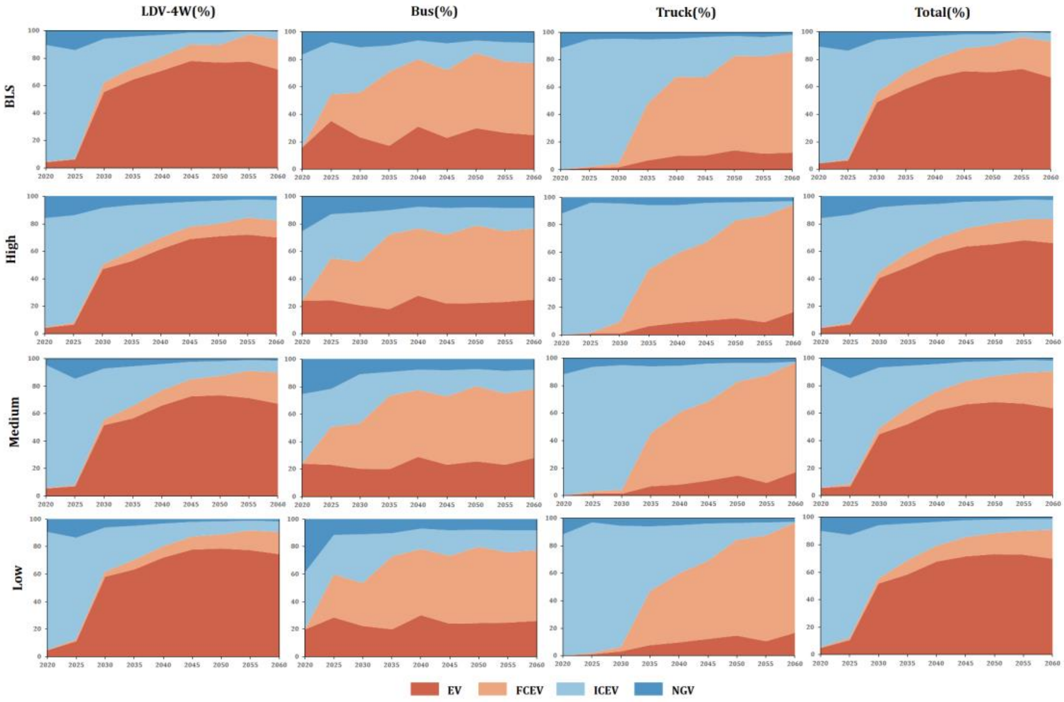

Figure S25. Vehicle penetration rate from 2020 to 2060 in which EVs are equipped with NCM9.5.5 LIBs. The abbreviations of EV, FCEV, ICEV, HEV, NGV, and LDV-4W are the same as in Fig. S1; The scenario of BLS is the same as in Fig. S5; The scenarios of High, Medium, and Low are the same as in Fig. S3.

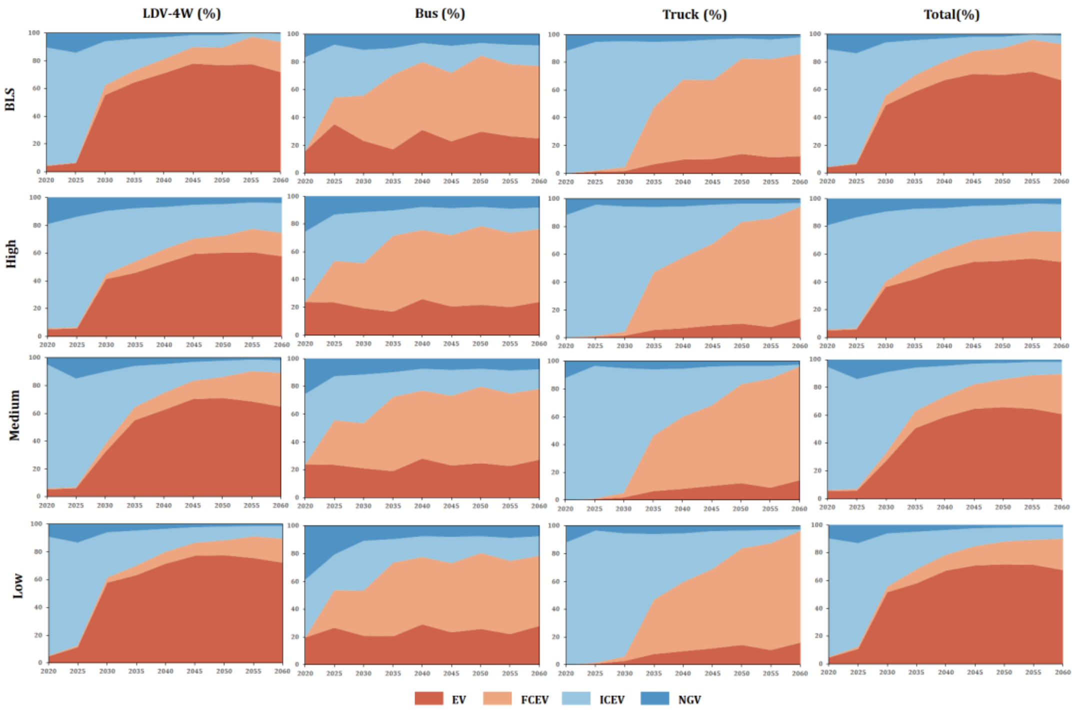

Figure S26. Vehicle penetration rate from 2020 to 2060 in which EVs are equipped with NCA LIBs. The abbreviations of EV, FCEV, ICEV, HEV, NGV, and LDV-4W are the same as in Fig. S1; The scenario of BLS is the same as in Fig. S5; The scenarios of High, Medium, and Low are the same as in Fig. S3.

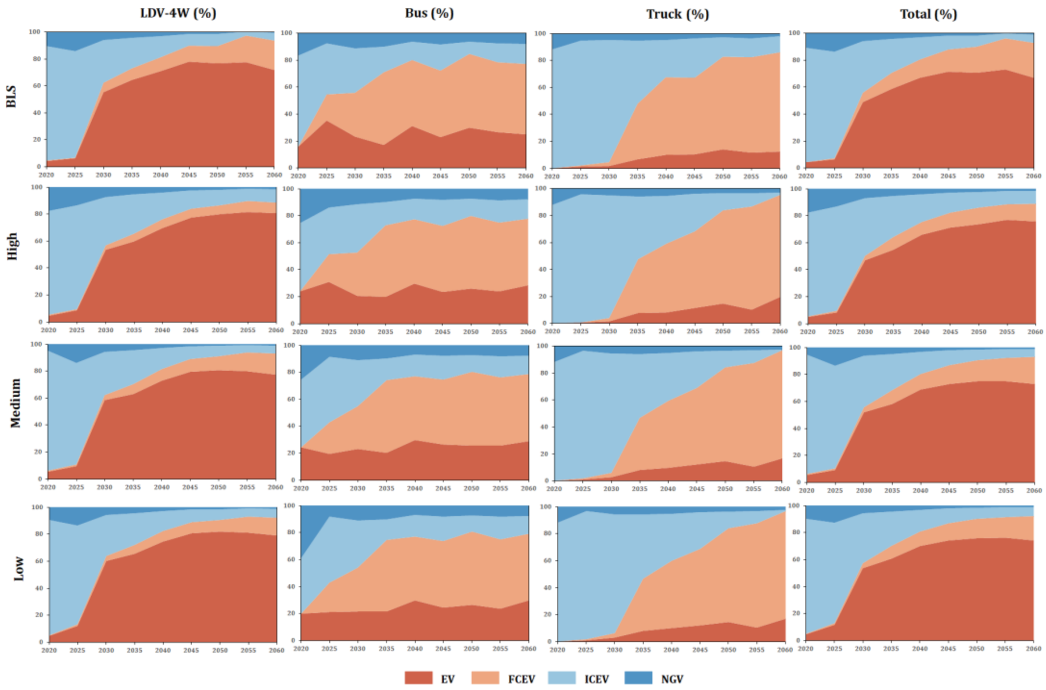

Figure S27. Vehicle penetration rate from 2020 to 2060 in which EVs are equipped with LFP LIBs. The abbreviations of EV, FCEV, ICEV, HEV, NGV, and LDV-4W are the same as in Fig. S1; The scenario of BLS is the same as in Fig. S5; The scenarios of High, Medium, and Low are the same as in Fig. S3.

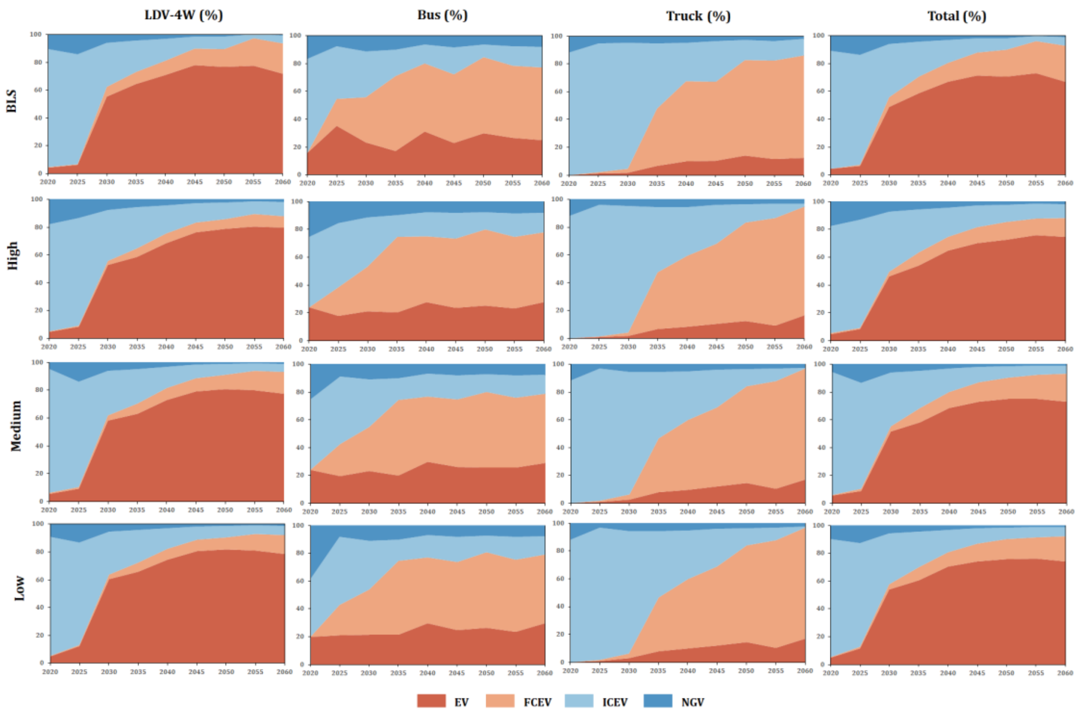

Figure S28. Vehicle penetration rate from 2020 to 2060 in which EVs are equipped with LMO LIBs. The abbreviations of EV, FCEV, ICEV, HEV, NGV, and LDV-4W are the same as in Fig. S1; The scenario of BLS is the same as in Fig. S5; The scenarios of High, Medium, and Low are the same as in Fig. S3.

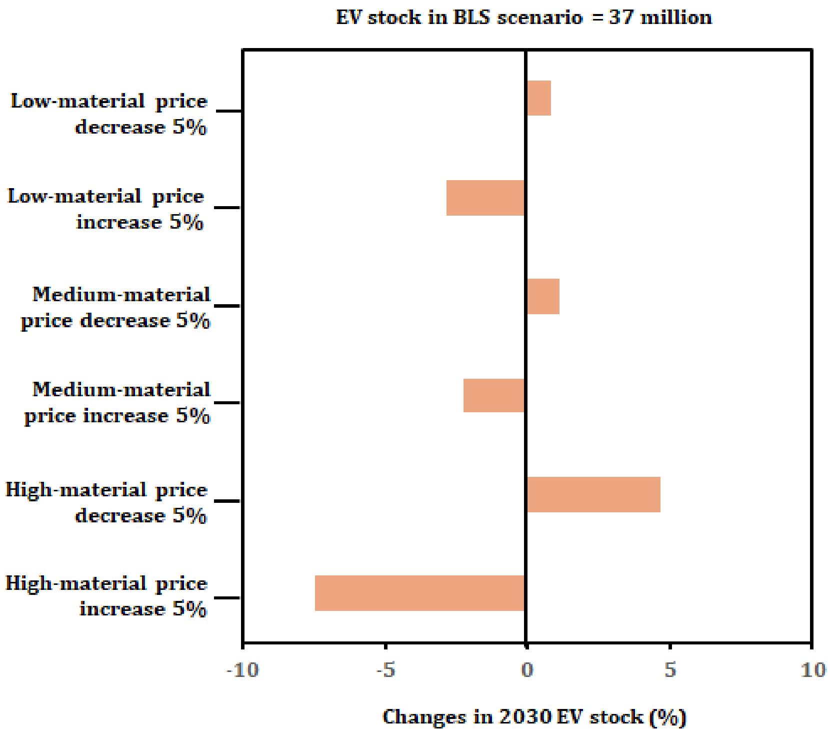

Figure S29. Sensitivity analysis for EV deployment in China in 2030. The abbreviation of EV is the same as in Fig. S1; EVs are equipped with NCM622 LIBs; The scenario of BLS is the same as in Fig. S5; The scenarios of High, Medium, and Low are the same as in Fig. S3.

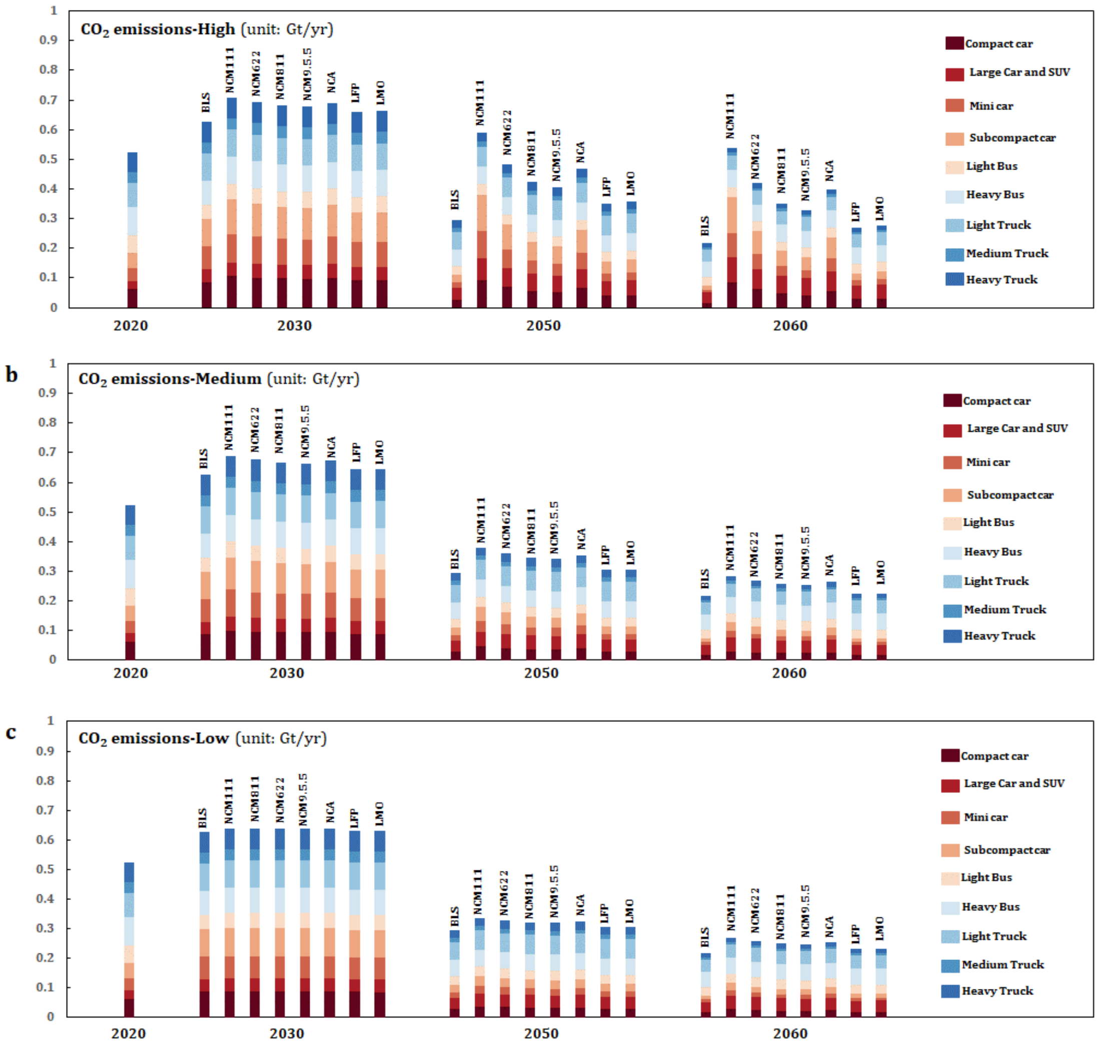

Figure S30. CO_2 emissions by sector in road transportation from 2020 to 2060. The scenario of BLS is the same as in Fig. S5; The scenarios of High, Medium, and Low are the same as in Fig. S3.

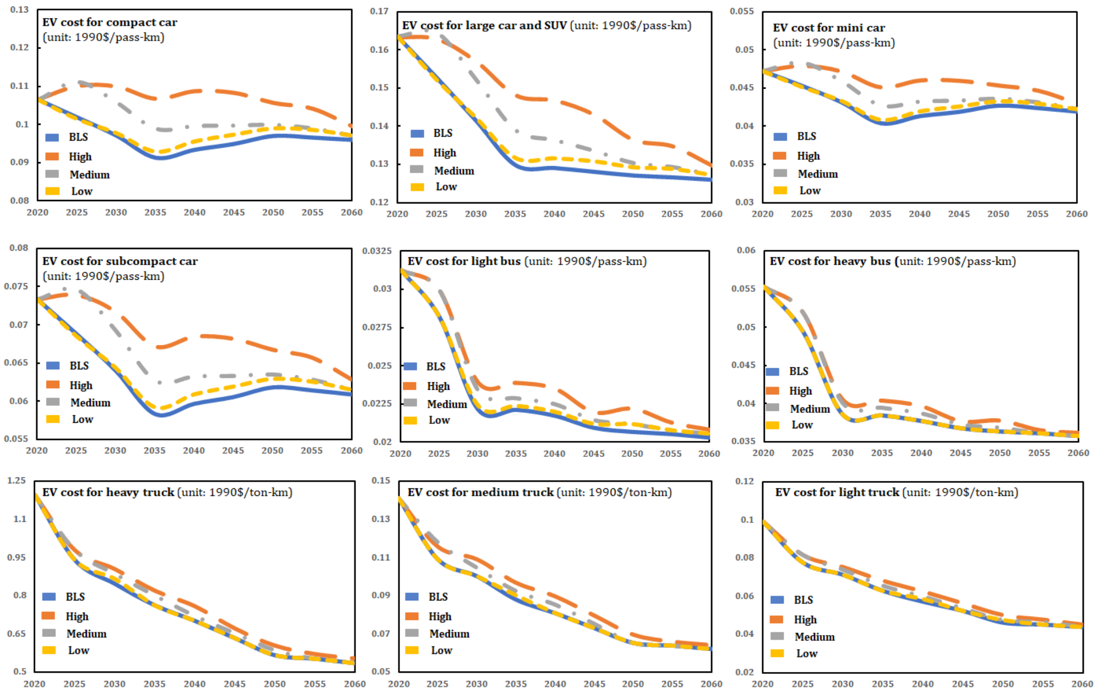

Figure S31. Cost evolution of EV equipped with NCM111 LIBs from 2020 to 2060 in recycling scenario. The scenario of BLS is the same as in Fig. S5; The scenarios of High, Medium, and Low are the same as in Fig. S3; The abbreviation of EV is the same as in Fig. S1.

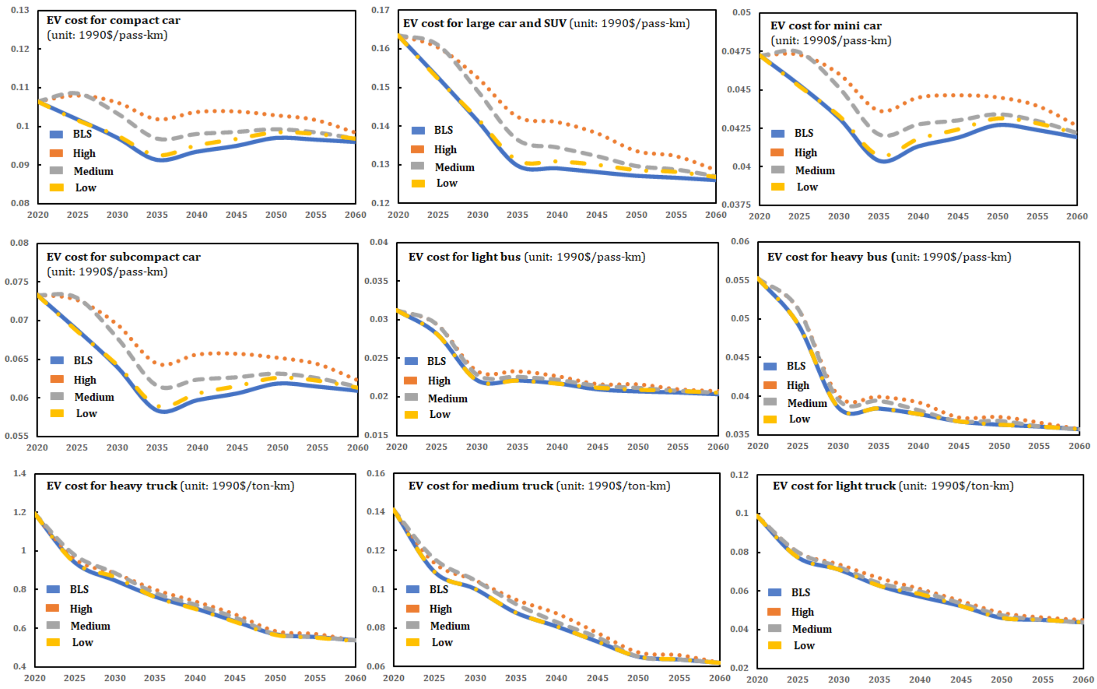

Figure S32. Cost evolution of EV equipped with NCM622 LIBs from 2020 to 2060 in recycling scenario. The scenario of BLS is the same as in Fig. S5; The scenarios of High, Medium, and Low are the same as in Fig. S3; The abbreviation of EV is the same as in Fig. S1.

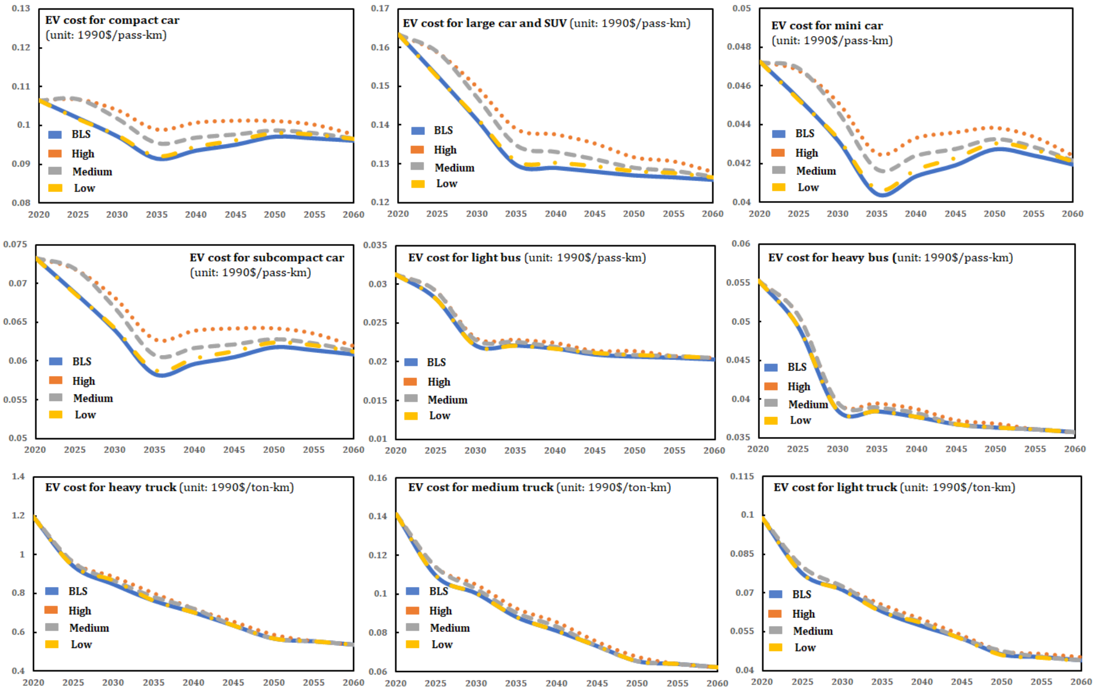

Figure S33. Cost evolution of EV equipped with NCM811 LIBs from 2020 to 2060 in recycling scenario. The scenario of BLS is the same as in Fig. S5; The scenarios of High, Medium, and Low are the same as in Fig. S3; The abbreviation of EV is the same as in Fig. S1.

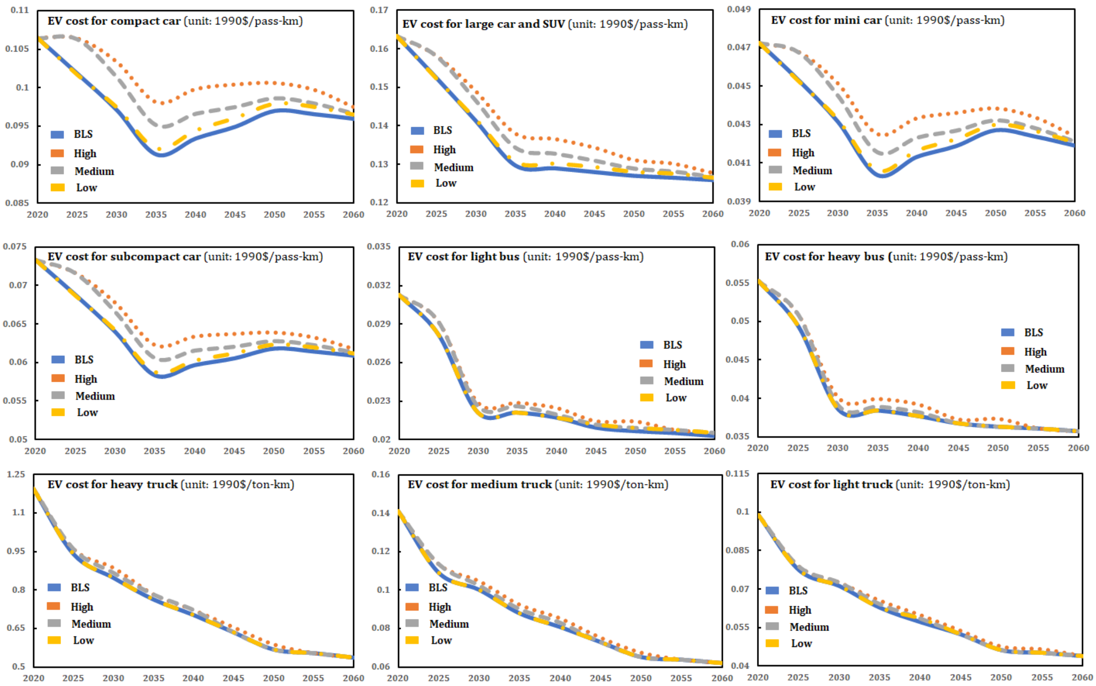

Figure S34. Cost evolution of EV equipped with NCM9.5.5 LIBs from 2020 to 2060 in recycling scenario. The scenario of BLS is the same as in Fig. S5; The scenarios of High, Medium, and Low are the same as in Fig. S3; The abbreviation of EV is the same as in Fig. S1.

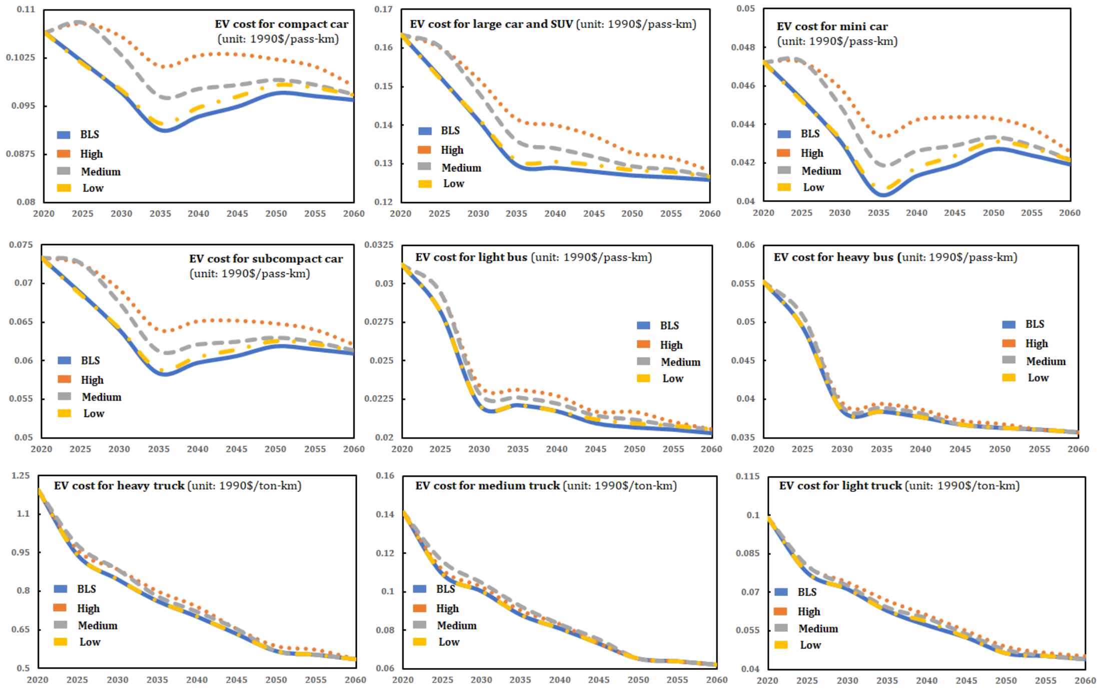

Figure S35. Cost evolution of EV equipped with NCA LIBs from 2020 to 2060 in recycling scenario. The scenario of BLS is the same as in Fig. S5; The scenarios of High, Medium, and Low are the same as in Fig. S3; The abbreviation of EV is the same as in Fig. S1.

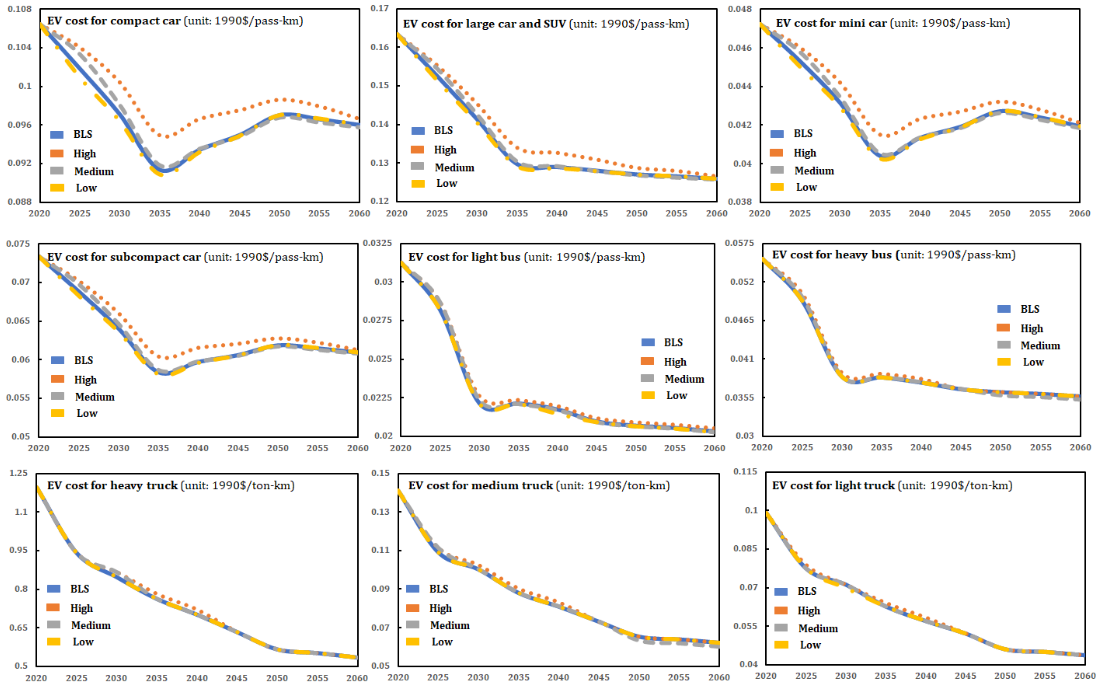

Figure S36. Cost evolution of EV equipped with LFP LIBs from 2020 to 2060 in recycling scenario. The scenario of BLS is the same as in Fig. S5; The scenarios of High, Medium, and Low are the same as in Fig. S3; The abbreviation of EV is the same as in Fig. S1.

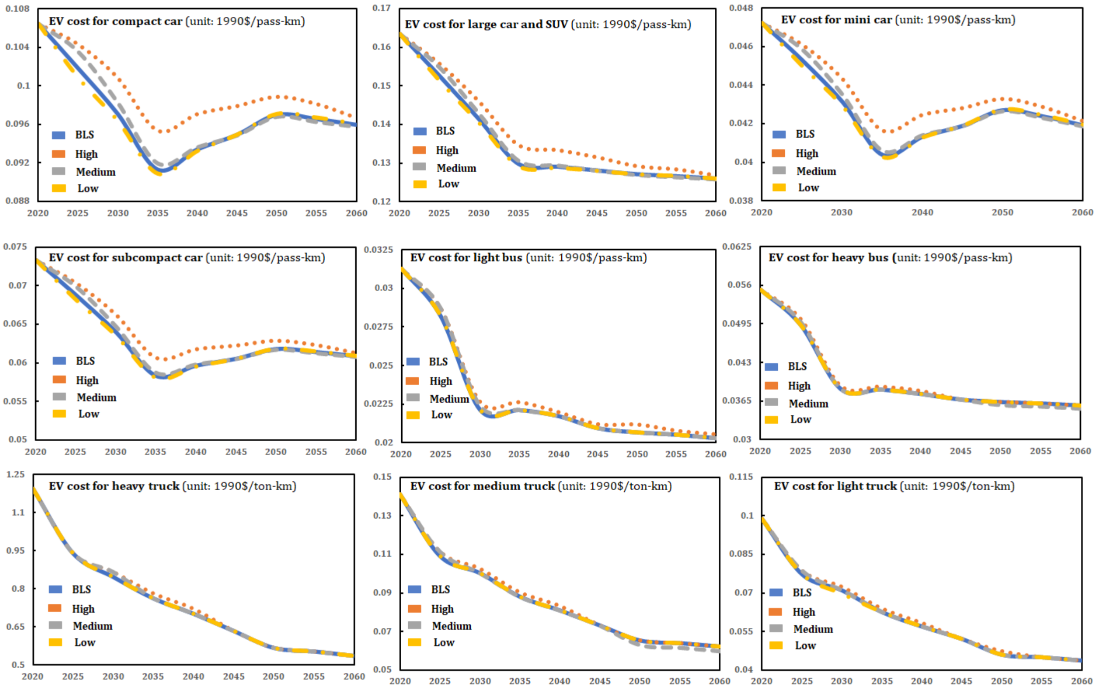

Figure S37. Cost evolution of EV equipped with LMO LIBs from 2020 to 2060 in recycling scenario. The scenario of BLS is the same as in Fig. S5; The scenarios of High, Medium, and Low are the same as in Fig. S3; The abbreviation of EV is the same as in Fig. S1.

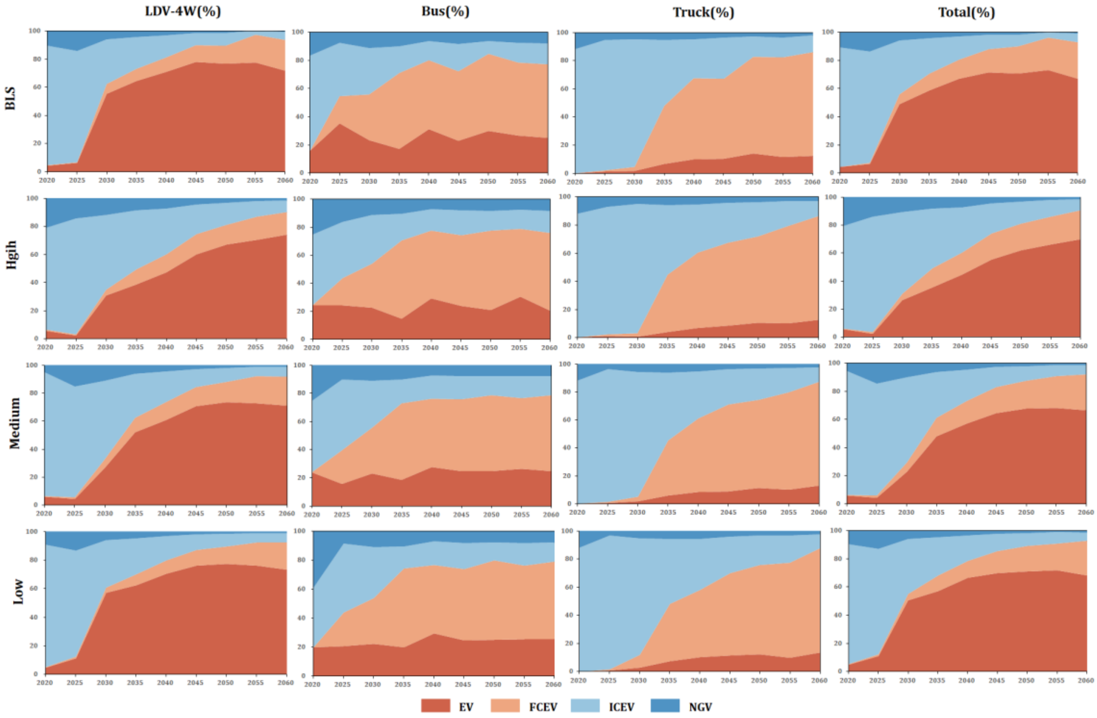

Figure S38. Vehicle penetration rate from 2020 to 2060 in recycling scenario in which EVs are equipped with NCM111 LIBs. The abbreviations of EV, FCEV, ICEV, HEV, NGV and LDV-4W are the same as in Fig. S1; The scenario of BLS is the same as in Fig. S5; The scenarios of High, Medium, and Low are the same as in Fig. S3.

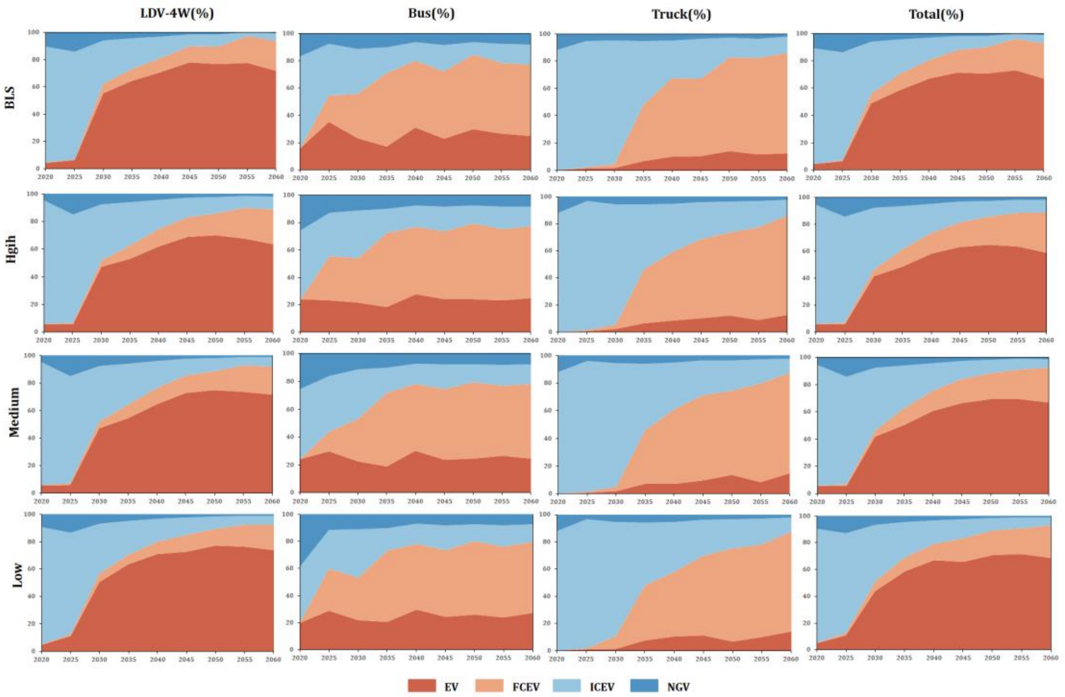

Figure S39. Vehicle penetration rate from 2020 to 2060 in recycling scenario in which EVs are equipped with NCM622 LIBs. The abbreviations of EV, FCEV, ICEV, HEV, NGV and LDV-4W are the same as in Fig. S1; The scenario of BLS is the same as in Fig. S5; The scenarios of High, Medium, and Low are the same as in Fig. S3.

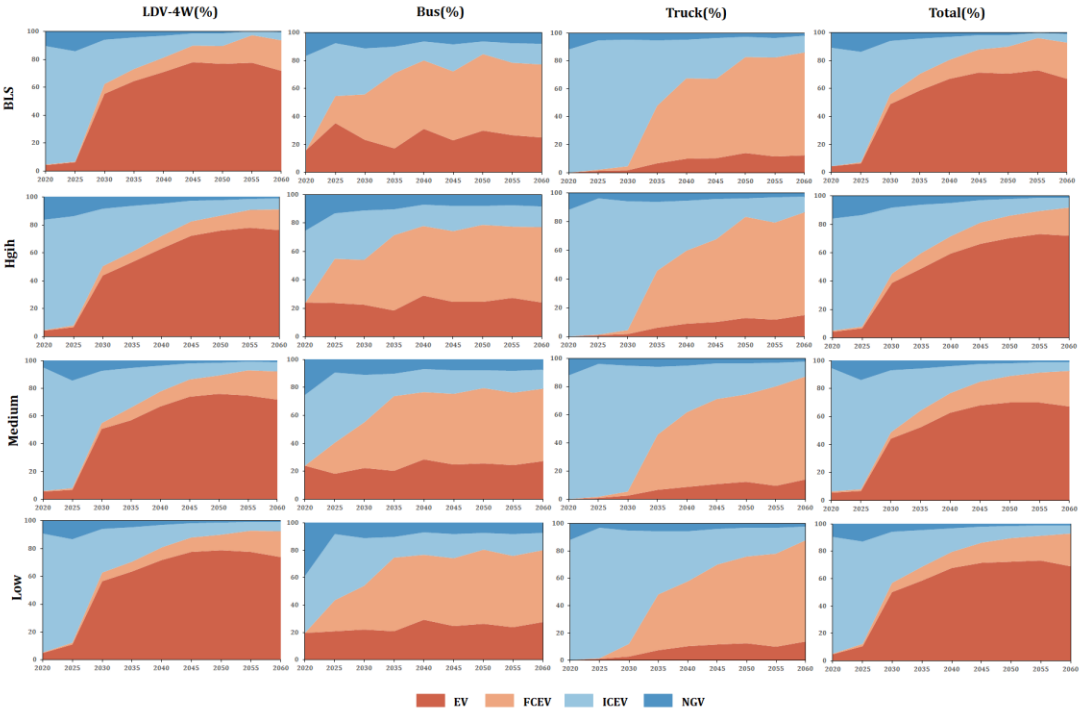

Figure S40. Vehicle penetration rate from 2020 to 2060 in recycling scenario in which EVs are equipped with NCM811 LIBs. The abbreviations of EV, FCEV, ICEV, HEV, NGV and LDV-4W are the same as in Fig. S1; The scenario of BLS is the same as in Fig. S5; The scenarios of High, Medium, and Low are the same as in Fig. S3.

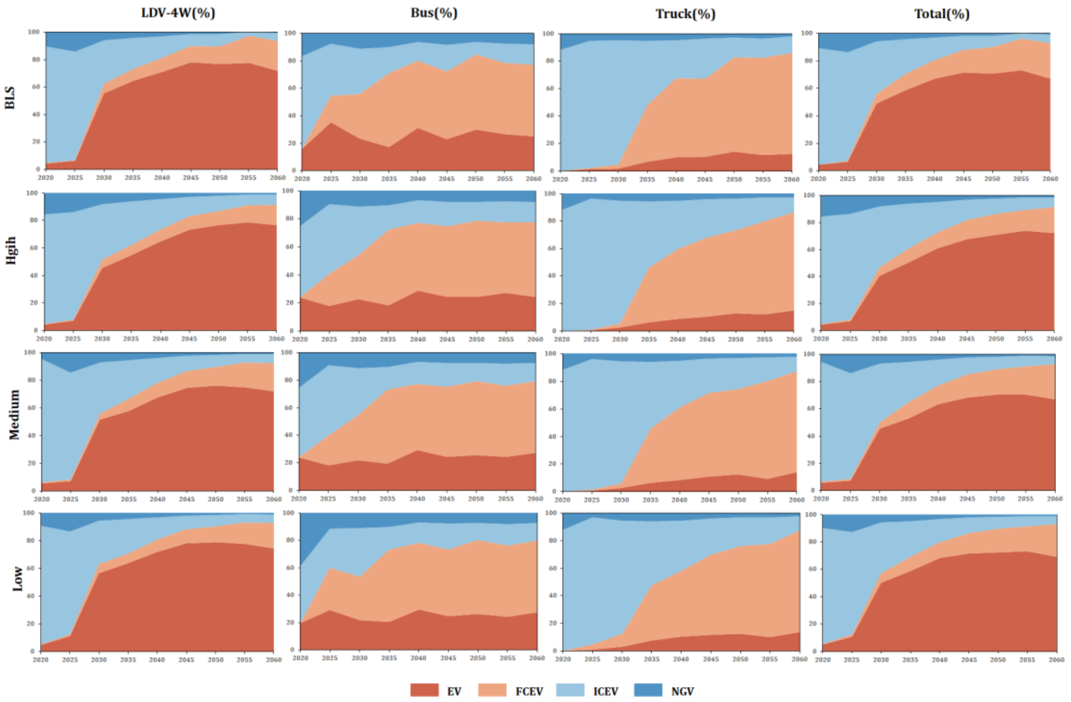

Figure S41. Vehicle penetration rate from 2020 to 2060 in recycling scenario in which EVs are equipped with NCM9.5.5 LIBs. The abbreviations of EV, FCEV, ICEV, HEV, NGV and LDV-4W are the same as in Fig. S1; The scenario of BLS is the same as in Fig. S5; The scenarios of High, Medium, and Low are the same as in Fig. S3.

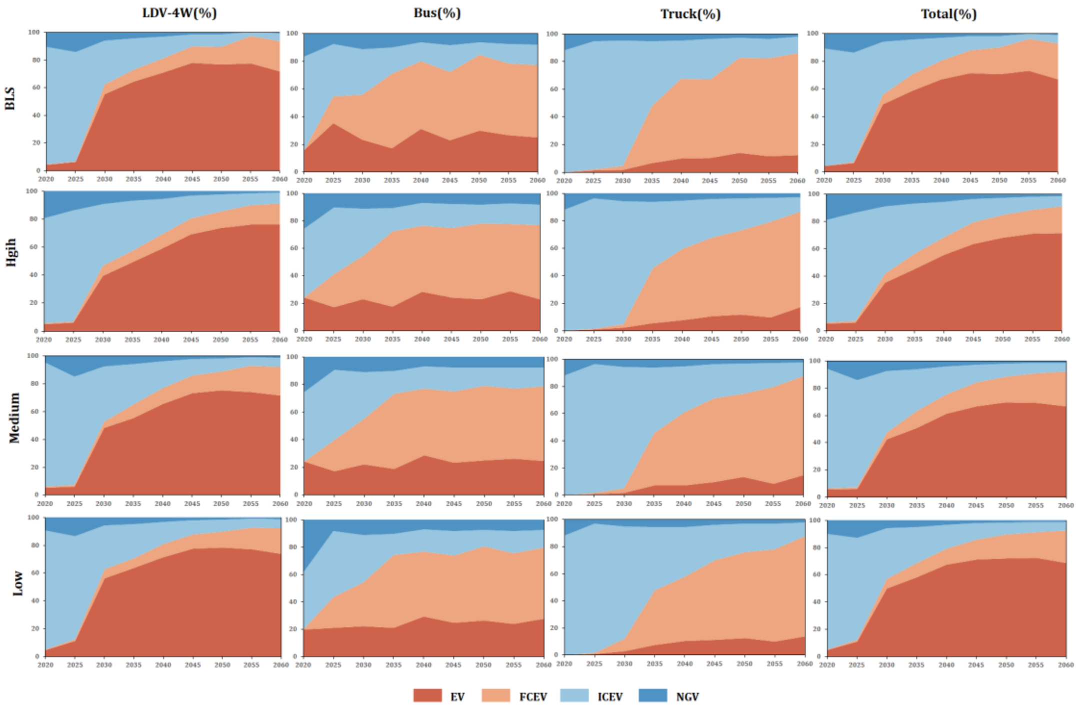

Figure S42. Vehicle penetration rate from 2020 to 2060 in recycling scenario in which EVs are equipped with NCA LIBs. The abbreviations of EV, FCEV, ICEV, HEV, NGV and LDV-4W are the same as in Fig. S1; The scenario of BLS is the same as in Fig. S5; The scenarios of High, Medium, and Low are the same as in Fig. S3.

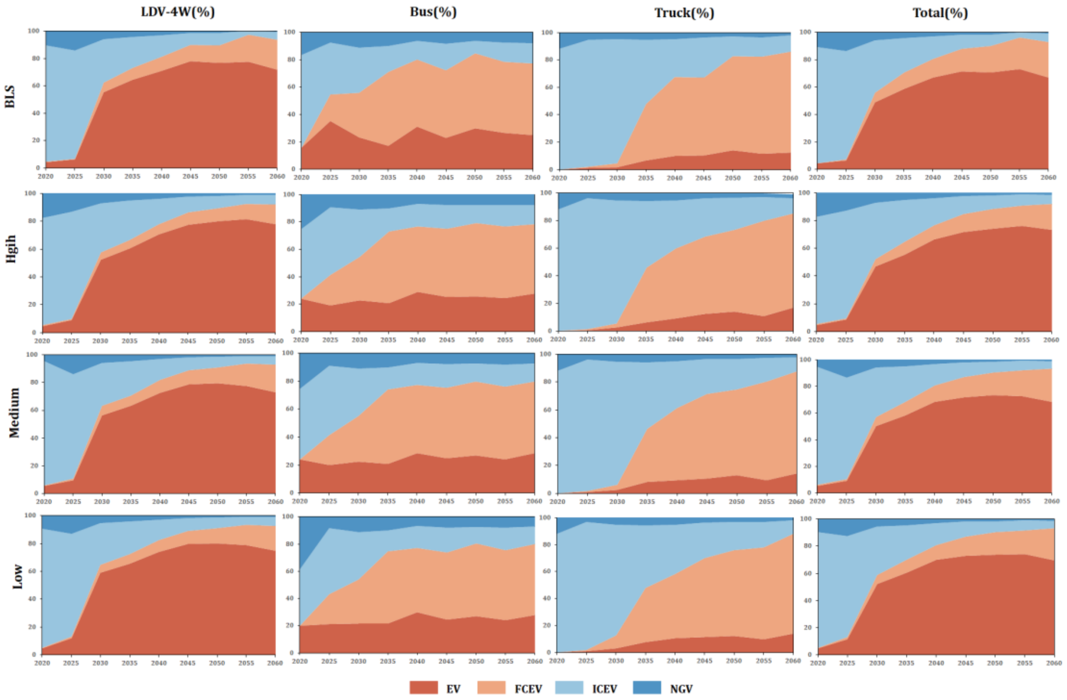

Figure S43. Vehicle penetration rate from 2020 to 2060 in recycling scenario in which EVs are equipped with LFP LIBs. The abbreviations of EV, FCEV, ICEV, HEV, NGV and LDV-4W are the same as in Fig. S1; The scenario of BLS is the same as in Fig. S5; The scenarios of High, Medium, and Low are the same as in Fig. S3.

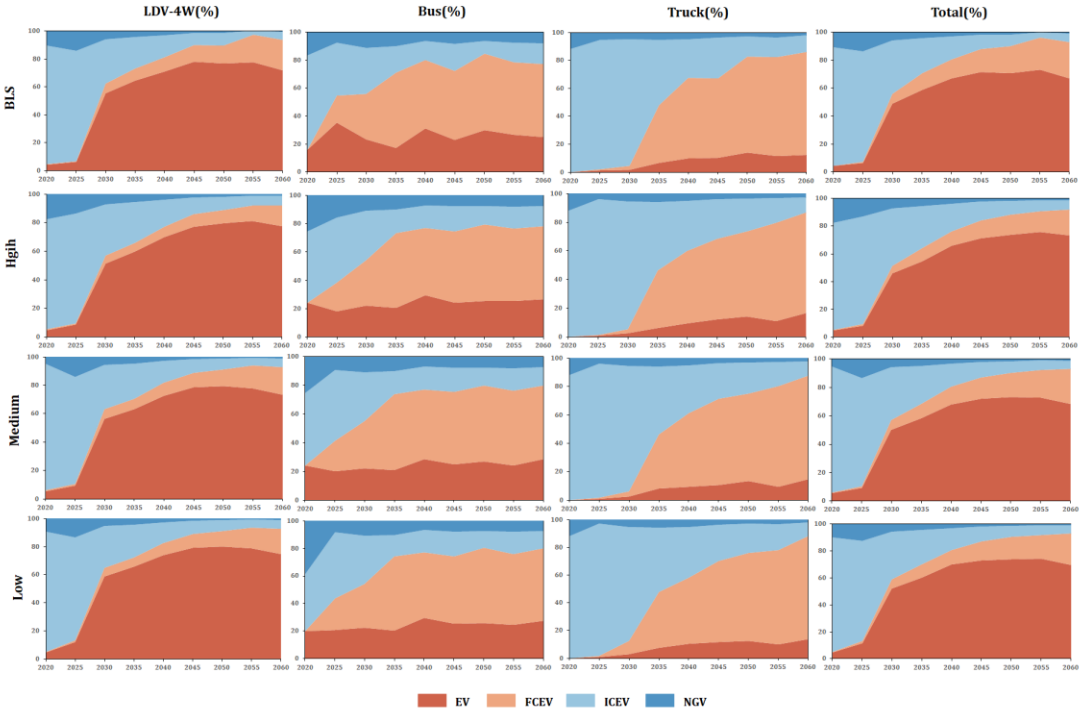

Figure S44. Vehicle penetration rate from 2020 to 2060 in recycling scenario in which EVs are equipped with LMO LIBs. The abbreviations of EV, FCEV, ICEV, HEV, NGV and LDV-4W are the same as in Fig. S1; The scenario of BLS is the same as in Fig. S5; The scenarios of High, Medium, and Low are the same as in Fig. S3.

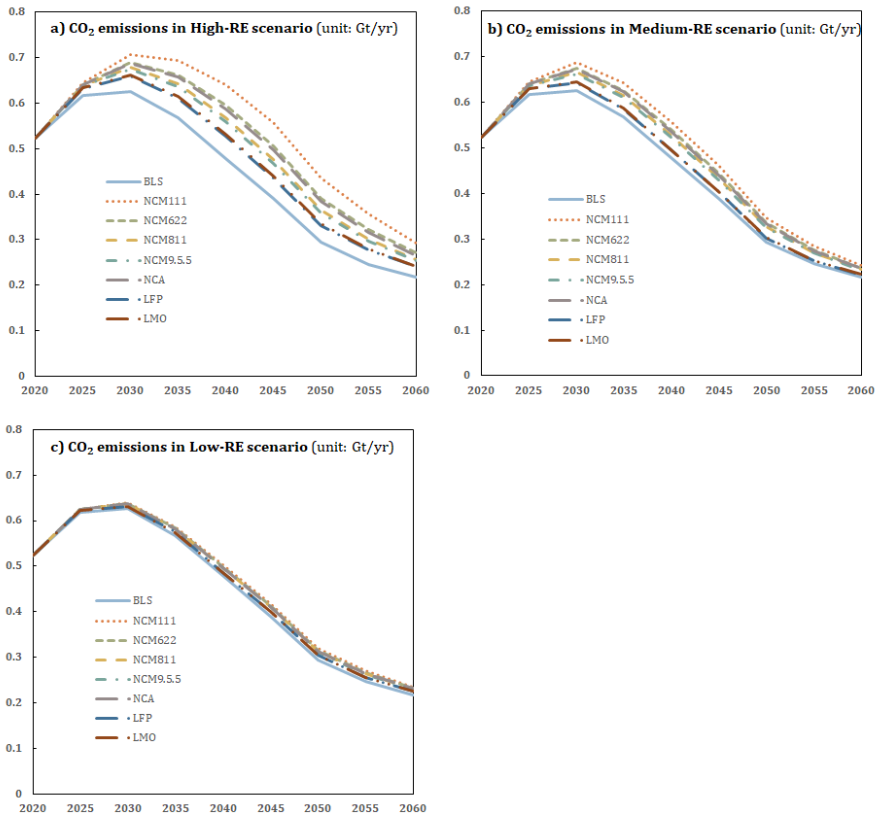

Figure S45. CO_2 emissions by year in recycling scenarios in road transportation from 2020 to 2060. The abbreviations of EV, FCEV, ICEV, HEV, and NGV are the same as in Fig. S1; The scenario of BLS is the same as in Fig. S5; The scenarios of High, Medium, and Low are the same as in Fig. S3; RE is a scenario in which only primary demand is affected by the market price of the material concerned.

References

1. Kim, S. H., Edmonds, J., Lurz, J., Smith, S. J. & Wise, M. The ObjECTS framework for integrated assessment: hybrid modeling of transportation. *Energy J.* (2006).
2. Kyle, P. & Kim, S. H. Long-term implications of alternative light-duty vehicle technologies for global greenhouse gas emissions and primary energy demands. *Energy Policy* **39**, 3012-3024 (2011).
3. Sun, Y., Delucchi, M. & Ogden, J. The impact of widespread deployment of fuel cell vehicles on platinum demand and price. *Int. J. Hydrogen Energy* **36**, 11116-11127 (2011).
4. MineDesign. Metal price forecasting. http://minewiki.engineering.queensu.ca/mediawiki/index.php/Metal_price_forecasting (2014).
5. Historical Monthly Consumer Price Index All Urban Consumers (CPI-U). *Bureau of Labor Statistics, Washington,* 2013).
6. Metalary. *Iron price*. https://www.metalary.com/iron-price/ (2019).
7. Infomine. *1 year aluminum prices and price charts*. http://www.infomine.com/investment/metal-prices/aluminum/1-year/ (2019).
8. Fickling, D. This cobalt chemistry experiment is rich with variables. https://www.bloomberg.com/gadfly/articles/2017-09-28/cobalt-s-chemistry-experiment (2017).
9. Leader, A., Gaustad, G. & Babbitt, C. The effect of critical material prices on the competitiveness of clean energy technologies. *Mater. Renewable Sustainable Energy* **8**, 1-17 (2019).
10. Wang, P., Chen, L.-Y., Ge, J.-P., Cai, W. & Chen, W.-Q. Incorporating critical material cycles into metal-energy nexus of China's 2050 renewable transition. *Appl. Energy* **253**, 113612 (2019).
11. Hao, H. *et al.* Impact of transport electrification on critical metal sustainability with a focus on the heavy-duty segment. *Nat. Commun.* **10**, 1-7 (2019).
12. Abdelbaky, M., Peeters, J. R. & Dewulf, W. On the influence of second use, future battery technologies, and battery lifetime on the maximum recycled content of future electric vehicle batteries in Europe. *Waste Manage.* **125**, 1-9 (2021).
13. Patry, G., Romagny, A., Martinet, S. & Froelich, D. Cost modeling of lithium-ion battery cells for automotive applications. *Energy Sci. Eng.* **3**, 71-82 (2015).
14. CleanTeq. *Nickel & cobalt for lithium ion batteries*. http://www.cleanteq.com/wp-content/uploads/2017/12/171205_Clean-Teq-LITHIUM-ION-BATTERIES.pdf (2017).
15. Chung, D., James, T., Elgqvist, E., Goodrich, A. & Santhanagopalan, S. *Automotive Lithium-ion Battery (LIB) Supply Chain and US Competitiveness Considerations*. (National Renewable Energy Lab.(NREL), Golden, CO (United States), 2015).
16. Olivetti, E. A., Ceder, G., Gaustad, G. G. & Fu, X. Lithium-ion battery supply chain considerations: analysis of potential bottlenecks in critical metals. *Joule* **1**, 229-243 (2017).
17. USGS. Lithium. https://www.usgs.gov/centers/nmic/lithium-statistics-and-information (2019).
18. InvestmentMine. *Commodity and Metal Prices, Metal Price Charts*. http://www.infomine.com/investment/metal-prices/ (2019).
19. Metalary. *Manganese price*. https://www.metalary.com/manganese-price/ (2019).
20. Satyapal, S. Us department of energy hydrogen and fuel cell overview. *US Dep. Energy EERE* (2016).

21. Arnold, R. *The Lowdown on Hydrogen—Part 1: Transportation*. (Energypost, 2017).
22. IOP Institute of Physics. *Fuel cells*. http://www.iop.org/resources/topic/archive/fuel/index.html (2019).
23. Marcinkoski, J., Spendelow, J. *DOE fuel cell technologies program record*. (U.S. Department of Energy, 2019).
24. Bustamante, M. L. & Gaustad, G. Challenges in assessment of clean energy supply-chains based on byproduct minerals: A case study of tellurium use in thin film photovoltaics. *Appl. Energy* **123**, 397-414 (2014).
25. Chemistry Views. *Fuel cell capacity and cost trends*. https://www.chemistryviews.org/details/ezine/4817371/Fuel_Cell_Capacity_and_Cost_Trends/ (2013).
26. Piccirilli, D. *Fact sheet—fuel cells*. https://www.eesi.org/papers/view/fact-sheet-fuel-cells (2015).
27. Ou, S. *et al.* Intensity and daily pattern of passenger vehicle use by region and class in China: estimation and implications for energy use and electrification. *Mitig. Adapt. Strateg. Glob. Chang.* **25**, 307-327 (2020).
28. Zheng, B. *et al.* How will greenhouse gas emissions from motor vehicles be constrained in China around 2030? *Appl. Energy* **156**, 230-240 (2015).
29. Bloomberg New Energy Finance. *Lithium price spike has moderate effect on batteries*. https://about.bnef.com/blog/lithium-price-spike-has-moderate-effect-on-batteries/ (2017).
30. Liu, B. *et al.* The impacts of critical metal shortage on China's electric vehicle industry development and countermeasure policies. *Energy* **248**, 123646 (2022).
31. Milovanoff, A., Posen, I. D. & MacLean, H. L. Electrification of light-duty vehicle fleet alone will not meet mitigation targets. *Nat. Clim. Change* **10**, 1102-1107 (2020).
32. McCollum, D. L. *et al.* Interaction of consumer preferences and climate policies in the global transition to low-carbon vehicles. *Nat. Energy* **3**, 664-673 (2018).
33. Isik, M., Dodder, R. & Kaplan, P. O. Transportation emissions scenarios for New York City under different carbon intensities of electricity and electric vehicle adoption rates. *Nat. Energy* **6**, 92-104 (2021).
34. Baars, J., Domenech, T., Bleischwitz, R., Melin, H. E. & Heidrich, O. Circular economy strategies for electric vehicle batteries reduce reliance on raw materials. *Nat. Sustainability* **4**, 71-79 (2021).
35. Peng, T., Ou, X., Yuan, Z., Yan, X. & Zhang, X. Development and application of China provincial road transport energy demand and GHG emissions analysis model. *Appl. Energy* **222**, 313-328 (2018).
36. Pan, X., Wang, H., Wang, L. & Chen, W. Decarbonization of China's transportation sector: in light of national mitigation toward the Paris Agreement goals. *Energy* **155**, 853-864 (2018).
37. Khanna, N., Lu, H., Fridley, D. & Zhou, N. Near and long-term perspectives on strategies to decarbonize China’s heavy-duty trucks through 2050. *Sci Rep* **11**, 1-14 (2021).
